# Comparison of Whole Blood and Peripheral Blood Mononuclear Cell Gene Expression for Evaluation of the Perioperative Inflammatory Response in Patients with Advanced Heart Failure

**DOI:** 10.1371/journal.pone.0115097

**Published:** 2014-12-17

**Authors:** Galyna Bondar, Martin Cadeiras, Nicholas Wisniewski, Jetrina Maque, Jay Chittoor, Eleanor Chang, Maral Bakir, Charlotte Starling, Khurram Shahzad, Peipei Ping, Elaine Reed, Mario Deng

**Affiliations:** 1 University of California Los Angeles, Los Angeles, CA, United States of America; 2 Columbia University, New York, NY, United States of America; 3 East Carolina University, Greenville, NC, United States of America; San Diego State University, United States of America

## Abstract

**Background:**

Heart failure (HF) prevalence is increasing in the United States. Mechanical Circulatory Support (MCS) therapy is an option for Advanced HF (AdHF) patients. Perioperatively, multiorgan dysfunction (MOD) is linked to the effects of device implantation, augmented by preexisting HF. Early recognition of MOD allows for better diagnosis, treatment, and risk prediction. Gene expression profiling (GEP) was used to evaluate clinical phenotypes of peripheral blood mononuclear cells (PBMC) transcriptomes obtained from patients' blood samples. Whole blood (WB) samples are clinically more feasible, but their performance in comparison to PBMC samples has not been determined.

**Methods:**

We collected blood samples from 31 HF patients (57±15 years old) undergoing cardiothoracic surgery and 7 healthy age-matched controls, between 2010 and 2011, at a single institution. WB and PBMC samples were collected at a single timepoint postoperatively (median day 8 postoperatively) (25–75% IQR 7–14 days) and subjected to Illumina single color Human BeadChip HT12 v4 whole genome expression array analysis. The Sequential Organ Failure Assessment (SOFA) score was used to characterize the severity of MOD into low (≤ 4 points), intermediate (5–11), and high (≥ 12) risk categories correlating with GEP.

**Results:**

Results indicate that the direction of change in GEP of individuals with MOD as compared to controls is similar when determined from PBMC versus WB. The main enriched terms by Gene Ontology (GO) analysis included those involved in the inflammatory response, apoptosis, and other stress response related pathways. The data revealed 35 significant GO categories and 26 pathways overlapping between PBMC and WB. Additionally, class prediction using machine learning tools demonstrated that the subset of significant genes shared by PBMC and WB are sufficient to train as a predictor separating the SOFA groups.

**Conclusion:**

GEP analysis of WB has the potential to become a clinical tool for immune-monitoring in patients with MOD.

## Introduction

Heart failure (HF) affects more than 5 million people in the United States. Mechanical Circulatory Support (MCS) therapy is a current temporary strategy for patients with end-stage HF who are not candidates for heart transplantation. Because of the limited availability of donor organs and the approval of MCS for long term (destination) therapy, the use of MCS therapy has grown rapidly over the last 10 years. MCS therapy is considered in patients who are no longer responsive to medical treatment, patients that are not candidates for heart transplantation (as destination or lifetime therapy), patients who are awaiting a heart transplant and/or are becoming too sick due to progressive heart failure, and also in patients with HF in whom myocardial function is expected to return to normal in a short period of time (as a bridge to recovery) [1]. Outcomes after MCS therapy have significantly improved, yet 10–20% of patients die during the first year post MCS-implantation, usually from sepsis and Multi Organ Dysfunction Syndrome (MOD) [2]. MOD is linked to an altered immune response induced by the device and the surgical procedure, and is influenced by the preexisting HF [3] **** [4] **** [5]. Multiple abnormal immune functions describe the critically ill patient: aberrant systemic inflammatory response syndrome (SIRS), altered production of antibodies, abnormal lymphocyte response regulation, release of chemical mediators including cytokines, nitric oxide, endothelin, and prostaglandins [6] **** [7] [8].

Risk prediction tools are commonly used in clinical practice to estimate outcomes. Among them is the Sequential Organ Failure Assessment (SOFA) score, which is useful to predict MOD following MCS therapy. Mortality and length of stay in the ICU and the hospital can be estimated by cross-sectional values of initial score or highest score as well as temporal changes in the SOFA score. Survival is limited for MCS patients with MOD who have high SOFA scores [9]. Early recognition of MOD has important implications in diagnosis, treatment and risk stratification of patients with AdHF undergoing high risk cardiovascular interventions such as MCS, high risk cardiac revascularization, or valve replacement.

However, the SOFA score as well as all other currently available risk scoring tools for MOD ICU patients do not incorporate parameters of immune function, i.e. indicators of the inflammatory response, even though MOD is linked to an exaggerated leukocyte-mediated SIRS. Therefore, we propose a comprehensive evaluation of the immune response associated with MCS implantation and states of multiorgan injury. After completion of the human genome project, global (whole transcriptome) methods of gene expression profiling (GEP) of various tissues and blood cell types have become available for genome-wide evaluation of clinical phenotypes that can now be used to improve clinical evaluation in multiple disease settings. [10] [11] **** [12] [13] **** [14] [15]. We have previously developed a PBMC GEP test to rule out heart transplant rejection [27], implemented the test clinically, showed its clinical utility [43] and showed its potential to predict clinical events after heart transplantation [42], and demonstrated the feasibility of PBMC whole transcriptome GEP in AdHF-patients undergoing MCS-implantation [44]. In this setting, evaluation of the information contained in the PBMC transcriptome may provide a promising solution to this important missing point in the assessment of the critically ill patient.

**Figure 1:**
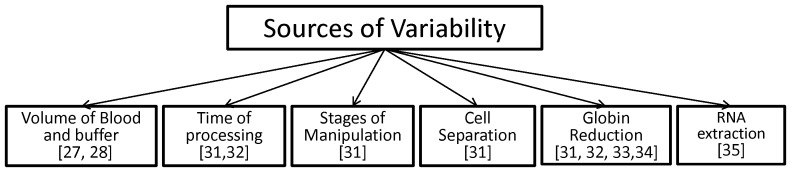
Variability of methodologies in the processing of gene expression in WB and PBMC.

In the ICU-setting, there are specific challenges to overcome in the development of novel genomic diagnostics. First, several methodological challenges apply to PBMC GEP, which may interfere with reproducibility through the addition of systematic bias when used in the setting of multicenter studies [6] [23] [24] [25] [26] [27] [28] [29]. Second, when approaching the evaluation of critically ill patients, the amount of blood that is obtained may add a complication to the process of recovery. PBMC processing for leukocyte isolation requires 8 ml of blood while WB methods require only 2.5 ml. These patients require multiple blood draws and efforts to minimize loss that is considered in the best interest of the patients. Third, these patients are exposed to blood transfusion, medications, and different degrees of oxygen saturation and fluid loading which could potentially cause error in the results. **(** **Fig. 1** **)**.

It is known that there is a significant overlap between WB and PBMC gene expression [16]. A study by Whitney et al (2003) found that the proportions of the variety of cell types in peripheral blood can affect the GEP of a patient and that these proportions can rapidly change after an inflammatory response [18]. Debey et al (2004) compared the effect of different isolation techniques in gene expression experiments and found that PBMC analysis yields a more sensitive diagnosis as compared to WB [19]. However, Freezor et al (2004) and Palmer et al (2006) discovered that the process was laborious and could affect the GEP [20] [21]. Most recently, Min et al (2012) evaluated the variability of GEP in human blood and lymphoblastoid cell lines. They showed a significant overlap in the gene expression profiles between WB and PBMC [22].

An important aspect concerning the applicability of new techniques that can be rapidly translated into clinical practice involves its feasibility, reproducibility, cost effectiveness, and ease of implementation, and application. For practical and methodological reasons, such studies are usually performed using purified PBMC samples. However, use of PBMC is labor-intensive and requires several methodological steps that must be strictly followed [16] **** [21] **** [23]. As an alternative, easy-to-use WB gene expression methods have been developed for clinical applications [30]. While comparisons have already been done for gene expression of PBMC and WB supporting the use of both methodologies, it is not known whether the same principles can be applied to critically ill patients undergoing complex surgical procedures [33]. These patients are usually exposed to multiple factors such as repeat blood transfusions, hemodilution, stressors and drugs which could interfere with the applicability of WB gene expression methodologies to this specific population.

Therefore, we hypothesize that mRNA profile of WB from patients with MCS can be used as a surrogate for mRNA profile of PBMC of patients with MCS to identify transcriptome fingerprints for biomarker discovery in various degrees of MOD [30], [31], [37].

## Methods

### Ethics statement

This study was approved by the Columbia University Office of Human Research Protection Program IRB # 2206 and all patients signed an informed consent.

### Patients

We collected blood samples from 31 consecutive HF patients (57±15 years old) undergoing cardiothoracic surgery (n = 29 MCS, n = 1 coronary artery bypass surgery (CABG) + aortic valve replacement (AVR), n = 1 transcatheter aortic valve replacement (TAVR) and 7 healthy age-matched controls). Samples were obtained between March 2010 and May 2011 at a single institution. PBMC was available for 30 patients and 7 controls, and WB was available for 31 patients and 3 controls. Main characteristics of the study samples are summarized in **Table 1**.

**Table 1:** Characteristics of the patients.

	Age	Days	SOFA	Gender
	median (Q1:Q3)			n (%)
**Control**	52(44.5:55.25)		0(0:0)	5(62.5)
**Low**	64(53:70.5)	12.5(18.75:8)	4(4:2.5)	13(92.9)
**Medium**	60.5(47.5:66.75)	7(9.75:7)	7(8.75:5.25)	5(50)
**High**	60.5(56.5:65.25)	8.5(20.25:4.5)	16.5(19:13.75)	3(37.5)

Calculations are based on all subjects participating in the study. There were 31 WB samples and 30 PBMC samples from patients and 7 and 3 PBMC and whole blood controls respectively.

Samples were collected at a single time-point postoperatively at a median of 8 days after device implantation (25–75% IQR 7 – 14 days). The variability of the day 8 sampling time point was dictated by the clinical circumstances. To assess for MOD, we utilized the SOFA score. The SOFA score is a simple, validated, and widely accepted tool that can be easily obtained at a patient's bedside and is used to assess patient's disease severity and predict survival in the critical care unit [40]. This score has been applied to different study populations including patients with MCS [41]. SOFA is an integer score that assigns a numerical variable to each of 6 major organ systems to quantify the severity of organ failure. Values range between 0 and 4. Each system's value is summed into a single SOFA score. Therefore the sum score ranges between 0 to 24 and correlates with the severity of MOD and clinical outcomes. Following our previous studies [42], [43], [44], [45], we divided the population into SOFA score risk subgroups which were defined as low (≤ 4 points), intermediate (5–11), and high (≥ 12). SOFA score was computed for each patient at the time that gene expression samples were obtained.

### Sample collection and RNA isolation

Blood was drawn and collected into PaxGene tubes (Becton Dickinson, Franklin Lakes, NJ) for the WB mRNA gene expression analysis or into a CPT tube (Becton Dickinson, Franklin Lakes, NJ) for PBMC cell isolation. For the WB samples, 2.5ml of blood were collected using PaxGene tubes. Samples were incubated at room temperature for 2 hours for RNA stabilization and then stored at −80°C. RNA was extracted from WB using the PAXgene Blood RNA Kit (Qiagen, Valencia, CA) according to the manufacturer's protocol which includes a globin reduction step. Briefly, samples were removed from −80°C and incubated at room temperature for 2 hours. Following lysis, the tubes were centrifuged for 10 min at 5,000×g, the supernatant was discarded and 500µL of RNase-free water was added to the pellet. Subsequently, the tubes were vortexed thoroughly to re-suspend the pellet, centrifuged for 10 min at 5000×g and the entire supernatant was discarded. The pellet was re-suspended in 360µL of buffer BR1 by vortexing and further purification of RNA was done following the manufacturer's protocol with on-column DNase digestion.

For the PBMC samples, mononuclear cells were isolated from 8 ml of blood collected by Vacutainer cell preparation tubes (CPT) with sodium citrate (Becton Dickinson, Franklin Lakes, NJ), resuspended in RNeasy Lysis Buffer (RLT, Qiagen, Valencia, CA) within 2 hours of phlebotomy. Total RNA was isolated from each sample (RNeasy, Qiagen, Valencia, CA). Quality of the purified RNA was verified on an Agilent 2100 Bioanalyzer (Agilent Technologies, Palo Alto, CA); RNA concentrations were determined using a NanoDrop ND-1000 spectrophotometer (NanoDrop Technologies, Wilmington, DE).

### Microarray data processing and analysis

After RNA extraction, quantification and quality assessment, total mRNA was amplified and hybridized on the Illumina single color Human BeadChip HT12 v4 whole genome expression array. Each array targets more than 47,000 probes derived from the National Center for Biotechnology Information Reference Sequence (NCBI) RefSeq Release 38 (November 7, 2009) and other sources. All samples were processed in a single core facility. After hybridization and microarray chip processing, feature extraction was carried out in the Illumina Beadarray platform (Illumina, San Diego, CA). Data was then subjected to quantile normalization using GenomeStudio (Illumina, San Diego, CA) and normalized expression files for PBMC and WB were used hereafter for comparison.

Filtering was done against background and only those probes having more than 20% variation in the gene expression were retained. Probes mapping to the same gene transcript were not averaged. Data was then transferred to GeneSpring GX 12 (Agilent, Palo Alto, CA). Sample data was normalized using the Quantile normalization method and quality control carried out as implemented in GeneSpring [46]. Given that the starting dataset showed a significant amount of noise in the signal for the WB samples, data was further assessed for the coefficient of variation for each entity within risk groups so only those with a coefficient lower than 0.75 in at least one group were kept for subsequent analysis. This criterion was employed to reduce the intra-group variability, thereby selecting the most reliable entities for potential biomarker candidates.

### Quantitative Real-time Polymerase Chain Reaction (RT-qPCR) and Validation

Microarray data were validated by Quantitative PCR on PBMC and WB obtained from an independent set of samples. Total RNA from PBMC cells were purified using RNeasy Mini Kit (Qiagen, Valencia, CA) and total RNA from WB cells were purified using the MagNA pure Compact System (Roche, Pleasanton, CA). CDNA was synthesized with iScript supermix for RT-qPCR (BioRad, Hercules, CA). RT-qPCR analysis was carried out with iTaq SYBR green supermix (BioRad, Hercules, CA) on the 7500 Fast Real-time PCR system (Applied Biosystems, Foster City, CA). 18S rRNA levels were used as an internal control for real-time PCR. Sequences of the primer pairs used were as follows: 18S rRNA, 5′-GTAACCCGTTGAACCCATT-3′ and 5′-CCATCCAATCGGTAGTAGCG-3′; IL11RA, 5′-ACTTCCTGCTCAAGTTCCGT-3′ and 5′-GGCACTGACTCGTACAGCAT-3′; IL2RB, 5′-TTCTAGCGTCAGTGCTGGAG-3′ and 5′-CCTCAGAGATCCCAAAGGAA-3′; TRAF3IP3, 5′- GAGGCTCTGAAGGAGGACTG-3′ and 5′- TATCTGCTCCCTGCAGTTTG-3′; LAT2, 5′- CTACCCACCTGTCACCTCCT-3′ and 5′- CTGTTGGCACCATCAGAATC-3′; CCL5, 5′- AAGGAAGTCAGCATGCCTCT-3′ and 5′- TTTGCCAGTAAGCTCCTGTG-3′; GNLY, 5′-TTCTAGCGTCAGTGCTGGAG-3′ and 5′- ATGCCTTTCACACCCTGTTT-3′; MAP4K1, 5′- AAGATCCAGGACACCAAAGG-3′ and 5′- CTGGTACCACTGAAGCAGGA-3′; ABLIM1, 5′- GTGCAGTTCCCATGAGTCAC-3′ and 5′- GGACAATGGTTTCCTCTGCT-3′; CD96, 5′- AACACCATGGCTGTCACACT-3′ and 5′- AGGCTCGATGGTTCTCAACT-3′; GRB10, 5′- GCAGCCAGTCAGTCTTTCAA-3′ and 5′- GCAGCCAGTCAGTCTTTCAA-3′.

### Statistical analysis of gene expression

Normalized gene expression samples of normal controls were used as the reference against which samples from patients with low, medium, and high SOFA scores were compared. Statistical comparison was done by the Kruskal-Wallis analysis of variance method and corrected for multiple hypotheses testing using the Benjamini-Hochberg method. Genes that satisfied the significance criteria of less than 5% false discovery rate (FDR) were further analyzed for the enrichment of gene ontology (GO) and subjected to pathway analysis. For the GO analysis, we used High-Throughput GoMiner (HTGM) [47] (http://discover.nci.nih.gov/) to analyze the enrichment of GO categories by up- and down-regulated genes for both WB and PBMC [48]. HTGM analyzes data from all microarrays in a study, provides diagnostics for data interpretation, and allows visualization in the form of clustered heat maps [49]. Normally, the input to HTGM consists of a total-genes file (representing the entire Microarray or a randomly generated whole genome seed) and a changed-genes file (representing the genes with altered expression) relevant to the study purpose. The output generated by HTGM includes a summary of the results, a matrix whose rows are categories and whose columns are names of changed gene for hierarchical clustering of experiments and categories, and a statistical summary for each category including one-sided Fisher exact p-value and an FDR. Hierarchical clustering of enriched categories and changed genes allows determining which categories achieved statistical significance by virtue of containing essentially the same set of changed genes. For pathway analysis, we used the algorithm incorporated in Genespring, testing for differentially expressed genomic pathways based on the available repositories including Biopax, Wiki, and Reactome. A pathway list was obtained based on Entrez Gene ID and UniGene ID.

To assess the performance of PBMC and WB for biomarker development, we examined the accuracy of predicting the high-risk SOFA group subjects across data sets using the Support Vector Machine (SVM) Class Prediction module in GeneSpring GX 12 (Agilent, Palo Alto, CA), we trained linear kernel SVMs on the PBMC and WB gene datasets defined by the significance criteria described above, as well as the gene set defined by the intersection of these two sets and evaluated the misclassification rates between SOFA groups when each of these trained SVMs were applied to either dataset in its entirety.

## Results

### PBMC versus WB Differential Gene Expression (HF versus Controls)

**Figure 2:**
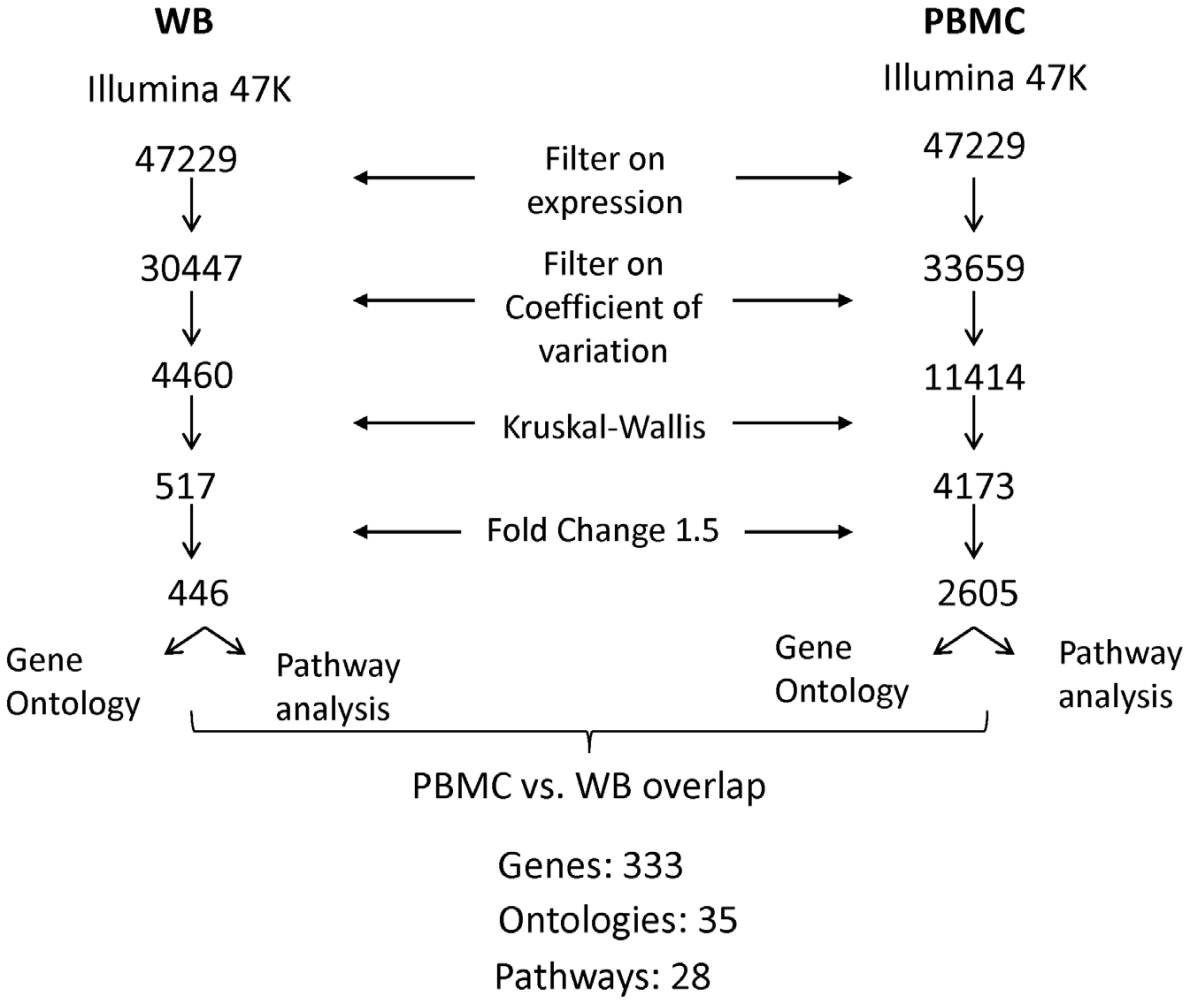
Flowchart of data analysis.

A general workflow of the analysis depicting differential gene expression for both groups is presented in **Fig. 2**.

The work presented here is the comparison between PBMC and WB whole transcriptome GEP and their correlation to the SOFA score. The combined risk groups of all 31 HF patients exhibited 2605 gene transcripts (2305 genes differentially expressed) in the PBMC and 446 (400 genes differentially expressed) in the WB dataset that had at least a 1.5 fold change in their expression level compared to healthy age-matched controls. While substantially more gene transcripts were differentially expressed in the PBMC compared to WB, there was a significant overlap among them with 333 genes (83.2%) shared by WB and PBMC. The direction of gene expression between WB and PBMC was consistent for 229 genes (68.7%) in the low risk compared to the control group, 245 genes (73.5%) in the medium risk group, and 251 genes (75.4%) in the high risk group. A list of the highest differentially expressed genes overlapping in WB and PBMC is provided in **Table 2**. A list of the overlapping genes and their Pearson correlations with the SOFA score, in both WB and PBMC, is included in the **S2 Table**. A complete list of genes both overlapping and non-overlapping for each dataset is also provided in the **S1 Table**.

**Table 2:** Most relevant representatives of the overlapping up- and down-regulated genes expressed in PBMC and WB between patients after intervention.

Gene symbol	Corrected p-value	Low SOFA score		Medium SOFA score		High SOFA score	
		Fold Change (PBMC)	Regulation	Fold Change	Regulation	Fold Change	Regulation
DARC	0.043	−1.320	down	−2.191	down	−3.601	down
ENO2	0.007	−1.431	down	−1.735	down	−2.222	up
WDR45	0.047	1.409	up	1.801	up	1.833	up
D40LG	0.005	−2.066	down	−2.702	down	−3.653	down
CD8B	0.006	−1.563	down	−1.798	down	−2.359	down
LCK	0.005	−1.848	down	−2.431	down	−3.232	down
FCGBP	0.005	−2.514	down	−2.900	down	−4.858	down
IL2RB	0.005	−2.473	down	−2.879	down	−8.016	down
CCL5	0.005	−1.559	down	−1.975	down	−6.794	down
CD8A	0.005	−2.182	down	−3.299	down	−8.575	down
ICAM2	0.018	−1.637	down	−1.606	down	−2.065	down
CD79B	0.005	−1.642	down	−1.536	down	−3.059	down
HLA-DQA1	0.009	−1.778	down	−2.170	down	−6.841	down
CD3G	0.005	−1.719	down	−2.177	down	−3.780	down
CD3D	0.005	−2.027	down	−3.295	down	−7.713	down
IL32	0.005	−1.569	down	−2.172	down	−3.799	down
VSIG4	0.005	1.257	up	1.962	up	10.599	up
S1PR5	0.005	−2.610	down	−2.857	down	−7.143	down
CD3D	0.005	−2.026	down	−3.154	down	−7.734	down
ITM2A	0.005	−1.913	down	−2.792	down	−5.529	down
IL1R2	0.011	1.135	up	1.976	up	15.480	up
KLRD1	0.005	−1.941	down	−2.374	down	−6.028	down
CD79A	0.005	−1.842	down	−1.809	down	−3.061	down
CD3E	0.005	−1.837	down	−2.734	down	−4.568	down
PTGDS	0.009	−1.752	down	−2.099	down	−3.329	down
TLR9	0.045	−1.168	down	−1.240	down	−1.277	down
GZMB	0.006	−2.316	down	−2.226	down	−7.583	down
CX3CR1	0.007	−1.200	down	−1.012	down	−3.954	down
AHSP	0.014	23.029	up	28.623	up	64.306	up
C19orf2	0.016	−1.469	down	−1.476	down	−1.823	down
TRADD	0.006	−1.453	down	−1.795	down	−2.209	down
CD96	0.005	−2.256	down	−2.748	down	−4.935	down
IL11RA	0.006	−1.783	down	−2.298	down	−3.334	down
CD8A	0.006	−2.283	down	−3.234	down	−8.740	down
BCL2	0.005	−1.341	down	−1.854	down	−3.008	down
FCER1A	0.005	−2.269	down	−3.763	down	−10.177	down
GZMA	0.006	−2.062	down	−2.194	down	−7.656	down
IL7R	0.005	−2.319	down	−3.891	down	−7.479	down
FAM102A	0.005	−2.212	down	−2.908	down	−4.824	down
SPOCK2	0.005	−1.935	down	−3.351	down	−5.789	down
SBK1	0.005	−2.070	down	−2.698	down	−5.390	down
CXCR5	0.010	−1.858	down	−2.112	down	−2.693	down
IL7R	0.005	−2.418	down	−3.905	down	−8.217	down
CD247	0.005	−2.436	down	−3.467	down	−9.013	down
CD2	0.005	−2.245	down	−3.349	down	−7.121	down
GPR56	0.006	−2.228	down	−2.612	down	−9.470	down
GNLY	0.005	−2.189	down	−2.584	down	−9.530	down
MYC	0.022	−1.383	down	−1.770	down	−2.474	down
GNLY	0.005	−2.302	down	−2.738	down	−9.103	down
HP	0.011	3.279	up	4.755	up	6.795	up
STAT4	0.005	−2.161	down	3.066	down	5.837	down

### PBMC versus WB Biomarker Candidate Gene Discovery

**Figure 3:**
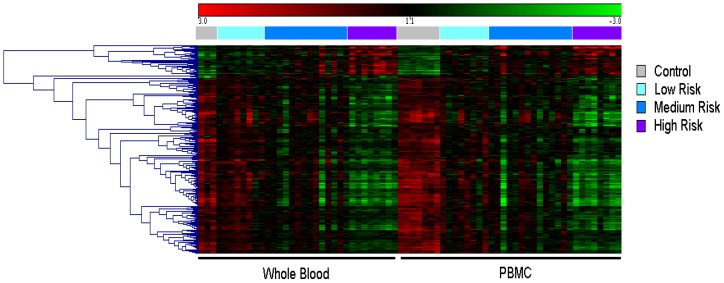
Clustered heatmap of the overlapping 333 genes in WB and PBMC shows highly correlated gene expression patterns.

Expression patterns of the highest differentially expressed genes have been clustered as a heatmap for visualization purposes and provided in **Fig. 3**.

This shows that patterns of the highest differentially expressed genes have similar profiles for PBMC and WB. For these genes, we used text mining algorithms as implemented in GeneSpring to identify those with the highest connectivity and most likely to have an important role in the inflammation process. These genes included IL11RA, CCLS, GNLY, MAP4K1, LAT2, CD96, GRB10, and IL2RB.

### Validation of PBMC and WB differentially regulated genes by RT-qPCR

To validate the microarray results in this study we used an alternative approach. We performed RT-qPCR to assay the 10 highest ranked genes, ranked first by statistical significance, secondly by correlation between WB and PBMC microarray expression levels, and finally by biological relevance **(** **Table 3** **).**

**Table 3:** Differentially expressed, highest connected genes sorted by connectivity (n).

Gene (Number of Connectivity)	Function	Disease
**IL11RA-** Interleukin 11 Receptor, Alpha	Stromal cell derived cytokine, uses gp130 transducing subunit in their high affinity receptors, members of the hematopoietic cytokine family.	Megakaryocytic leukemia
**IL2RB-** Interleukin 2 Receptor, Beta	T-cell mediated immune response. Intermediate and high affinity forms of IL2RB involved in receptor-mediated endocytosis and transduction of mitogenic signals from IL-2.	Occipital neuralgia, and granulomatous orchitis
**TRAF3IP3-** TRAF3 Interacting Jun N Terminal Kinase (JNK) Activating Modulator	Mediates cell growth through modulating c-Jun N-terminal kinase signal transduction pathway.	Rectum cancer, cerebral cavernous malformations 3.
**LAT2-** Linker for Activation of T-Cells	Encoded protein is phosphorylated by ZAP-70/Syk protein tyrosine kinases following activation of the T-Cell antigen receptor (TCR) signal transduction pathway. Protein then recruits multiplied adaptor proteins and downstream signaling molecules into multi-molecular signals.	Mitral valve stenosis, and alexia.
**CCL5-** Chemokine (C-C Motif) Ligand 5	Chemokine superfamily; member of the CC subfamily. Chemoattractant for blood monocytes, memory T helper cells and eosinophils. Causes histamine release from basophils and activates eosinophils. Cytokine is an HIV-suppressive factor produced by CD8+ cells.	Ulcer of lower limbs, meningoencephalitis.
**GNLY-** Granulysin	Saposin-like protein (SAPLIP). Present in cytotoxic granules of cytotoxic T-lymphocytes and NK cells. Has antimicrobial activity against M. Tuberculosis and other organisms.	Folliculitis, and spondylocostal dysostosis.
**MAP4K1-** Mitogen-Activated Protein Kinase Kinase Kinase	Protein coding. Gene associated with pancreatic cancer and pancreatitis.	Pancreatic cancer, and pancreatitis
**ABLIM1-** Actin binding LIM protein 1	Encodes a cytoskeletal LIM protein that binds to actin filaments via a domain that is homologous to erythrocyte dematin. LIM domains also function as protein binding interfaces, mediating specific protein-protein interactions.	Corneal neovascularization and alcohol dependence
**CD96-** CD96 Molecule	Type-1 membrane protein, immunoglobulin superfamily. Adhesive interactions of T-Cells and NK cells in late stage of immune response.	Cat eye syndrome and c-like syndrome
**GRB10-** Growth factor receptor bound protein 10	Small family of adapter proteins-interact with several receptor tyrosine kinases and signaling molecules. Encodes a growth factor receptor-binding protein that interacts with insulin receptors and insulin-like growth factor receptors.	Silver-russell syndrome, albright's hereditary osteodystrophy.

**Figure 4:**
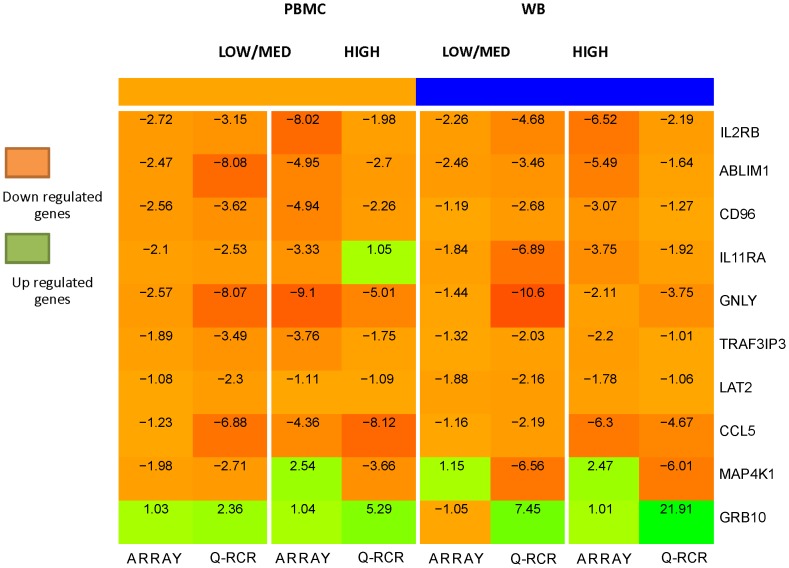
RT-qPCR validation of three differentially expressed genes within PBMC and WB subgroups, within SOFA groups. Darker colors correspond to higher magnitude of fold change. Fold changes showed similar patterns between all 10 genes, 9 of which are down-regulated, and 1 of which is up-regulated. 7 of 10 genes showed similar patterns between PBMC and WB and also within SOFA score groups according to our microarray and PCR results. 2 of 10 genes (IL11RA and GRB10) show an opposite result in HIGH PCR (PBMC) and low/med array (WB) correspondingly. Only 1 gene, MAP4K1, shows an opposite result between SOFA score groups.

To perform this validation, we analyzed RT-qPCR of 8 samples taken from across control, low/medium, and high SOFA score groups. Fold changes were calculated against control, averaged in each group by their logarithms, and transformed back. We compared the microarray and RT-qPCR results of expression of 10 genes between the SOFA score sub-groupings within the PBMC and WB groupings **(** **Fig. 4** **)**.

### PBMC versus WB Gene Ontology analysis

GO analysis identified 314 terms enriched by 1327 genes. 35 of these terms overlapped between WB and PBMC. GO categories included regulation of the immune response and related pathways including the innate and the adaptive immune responses. Interestingly, there was a pronounced enrichment of innate response GO terms by up-regulated genes and of the adaptive immune response by down-regulated genes as previously described in the setting of critical illness [16]. Other enriched GO terms were related to programmed cell death and the coagulation cascade, both well known to be involved in the biology of critical illness [17]. The identification of these processes in the context of the known biology supports the validity of our findings as opposed to random enrichment of GO categories. The overall enrichment found in the PBMC dataset compared to WB showed that the majority of genes, while different, were likely involved in similar biological processes. Thus, GO analysis suggests that for both PBMC and WB, expressed genes are involved in inflammation, apoptosis, and stress response related pathways among others. There is a significant difference in the number of GO categories but not in the processes represented by these categories. The lack of a significant enrichment of GO categories or members of a specific gene pathway in the WB dataset could be explained by different power assumptions when starting from a smaller number of genes. However, differences in the direction of gene expression could be explained by multiple mechanisms that reside in the interface between WB composition and PBMC and need to be further investigated. Genes in the overlap dataset include members of pathways such as Apoptosis (BCL2, MAGED1, and TNFRSF10B), Nephrin interactions (FYN), CD28 and CD28 co-stimulation (CD28r), Cell-extracellular matrix interactions (BCL2, LAT2, KLFR1, KLRD1, TRAT1, PTCRA, KLRB1, and ICAM2), Antiviral mechanism mediated by Interferon gamma signaling (IL1R2 and IRAK3), and Lipoprotein metabolism including LDL-mediated lipid transport, and Chylomicron-mediated lipid transport (LPL, VLDL, and TGIHDL). The most relevant GO categories enriched in PBMC and WB are described in **Table 4**.

**Table 4:** Most relevant representative of GO categories overlapping between PBMC and WB after intervention.

GO accession	GO Term	FDR	N selection	% selection	N Total	% Total
GO:0001817	regulation of cytokine production	0.061	37	1.4020462	125	0.747
GO:0002376	immune system process	0.000	243	9.208034	823	4.921
GO:0002682	regulation of immune system process	0.000	85	3.220917	257	1.537
GO:0006915	apoptosis	0.011	98	3.713528	425	2.541
GO:0010941	regulation of cell death	0.000	135	5.115574	545	3.259
GO:0042101	T cell receptor complex	0.000	11	0.41682455	12	0.072
GO:0045321	leukocyte activation	0.014	51	1.9325502	178	1.064
GO:0046649	lymphocyte activation	0.006	46	1.7430845	149	0.891

Of particular interest in the heatmap **(** **Fig. 3** **)** are the areas where multiple GO categories overlap or “cross-talk”, suggesting that mechanisms involved for a specific set of genes are common to different biological processes. Those GO terms were related to molecular mechanisms associated with inflammation leading to T-cell activation, activation of cytokine signaling cascades, and regulation of programmed cell death. Other important biological processes identified in the non-overlapping genes included “cross-talking” GO categories involving regulation of peptidase, caspase, and endopeptidase activity. A complete list of overlapping and non-overlapping GO categories is provided in **S3 Table**.

### PBMC versus WB Pathway analysis

The total number of differentially expressed pathways associated with up- or down-regulated genes was 26 in the WB dataset and 265 in the PBMC dataset. A total of 137 genes present in the WB pathway enrichment analysis were also included in the list of genes from PBMC pathways (466). The enrichment analysis revealed that 26 pathways were associated with up-regulated genes based on a corrected 2-tailed p-value <0.05 (5% FDR). Pathways differentially regulated in WB that were common to PBMC included the adaptive immune system. Out of 40 genes listed under the adaptive immune system pathway, 10 genes (25%) in the WB dataset were differentially expressed and 15 genes (37.5%) in the PBMC dataset were differentially expressed (p-values<0.001). Genes in this pathway including ITK, LAT, LCK, and TRAT1 were found to be up-regulated in both PBMC and WB. Eleven genes were up-regulated in PBMC but down-regulated in WB. The T-Cell receptor signaling pathway showed an enrichment of 30% (7/23 genes, (p =  3.8×10^−09^). Other important pathways included apoptosis, homeostasis, and those associated with HIV infections, CD28 and GPCR signaling. Significantly enriched pathways and corresponding gene expression are listed in **Table 5**.

**Table 5:** Pathway analysis by overlapping up- and down-regulated genes expressed in PBMC and WB after intervention.

Genes which overlap in PBMC and WB						
Pathways	have same direction within pathway		have opposite direction within pathway			
	PBMC and WB		PBMC		WB	
	down	up	down	up	down	up
Immune System	BTLA			TRAT1	TRAT1	
	LAT					
	PLCG1					
	PRKCQ					
	ZAP70					
Adaptive Immune System	ITK		BTLA	FYN	FYN	BTLA
	LAT		CARD11			CARD11
	LCK					
	TRAT1					
Immunoregulatory interactions between a Lymphoid and a non-Lymphoid cell	CD160		CD40LG	KLD1	KLD1	CD40LG
	CD96					
Costimulation by the CD28 family	ITK	LAT				
Chemokine receptors bind chemokines	CCL5					
	CCL7					
	CXCR6					
HIV Infection	LCK		CD247	NMT2	NMT2	CD247
Apoptosis	BCL2		GZMB	TRADD	TRADD	GZMB
	MAGED1			PRKCQ	PRKCQ	
	TNFRSF10B					
Class A/1 (Rhodopsin-like receptors)	CCR7		GPR18	CCL5	CCL5	GPR18
	CXCR6			CXR5	CXR5	
	GPR44					
Hemostasis	CD2			FYN	FYN	
	JAM3			LAT	LAT	
	KIFAP3					
	LCK					
	MAFG					
	PLCG1					
Nef and signal transduction	CD247					
	FYN					
Network Regulators	CD8A		CD3D	CD3G	CD3G	CD3D
Signal Transduction	PLCG1					
	SH3KBP1					
GPCR ligand binding	CXCR5		CCL5	CCR7	CCR7	CCL5
	GPR18			CXCR6	CXCR6	
	GPR44					
Signaling by GPCR	CXCR5	CCL5		CCR7	CCR7	
	CXCR6					
	GPR18					
	GPR44					
Signaling by Interleukins	IL1R2		IL2RB	IL7R	IL7R	IL2RB
	IRAK3					
Small Molecules	LCK			PRKCQ	PRKCQ	
				PLCG1	PLCG1	
				SIGIRR	SIGIRR	
TCR signaling	LAT			CARD11	CARD11	
	PLCQ1			ITK	ITK	
	PRKCQ					
	TRAT1					
	ZAP70					
The role of Nef in HIV-1 replication and disease pathogenesis	CD247		LCK			LCK
	FYN					
Toll Receptor Cascades	SIGIRR			IRAK3	IRAK3	
	TLR5					
Peptide ligand-binding receptors		CXR5		CCL5	CCL5	
				CCR7	CCR7	
				CXR6	CXR6	

For the pathway analysis, we found that of the 34 significant genes that overlapped between PBMC and WB pathway groups, 26 had concordantly regulated patterns in both groups. The other 8 genes showed an opposite expression in PBMC and WB datasets. Results observed in the WB samples were similar in several aspects to those in the PBMC datasets, supporting the role of these genes as potential biomarkers for early diagnosis and monitoring of MOD. Variations in the direction of gene expression suggest that perturbations in multiple signaling and cellular mechanisms occur in a comparable way in PBMC as in WB.

### Performance of classification using PBMC and WB

We employed a support vector machine (SVM) to examine the accuracy of predicting the high-risk SOFA group subjects across data sets. Misclassification rates between the SOFA groups, when each of these trained SVMs was applied to either dataset, are shown as confusion matrices in **Table 6**.

**Table 6:** SVM Misclassification Tables.

**A:** WB trained SVM applied to PBMC accurately predicts MED and HIGH, with confusion of CTRL and LOW.				
	**CTRL**	**LOW**	**MED**	**HIGH**
**CTRL**	1	6	0	0
**LOW**	2	5	1	0
**MED**	0	0	14	0
**HIGH**	0	0	0	8
**B:** PBMC trained SVM applied to WB is not good as expected, because the SVM was trained on PBMC genes that were not found to be significant in WB.				
**CTRL**	2	0	0	1
**LOW**	0	8	0	0
**MED**	3	0	10	1
**HIGH**	4	0	1	3
**C:** Overlapping PBMC SVM trained applied to PBMC (This means that the subset of genes in PBMC that are the overlapping genes with WB are sufficient in reproducing the prediction found by the entire set)				
**CTRL**	7	0	0	0
**LOW**	0	8	0	0
**MED**	0	0	14	0
**HIGH**	0	0	0	8
**D:** Overlapping PBMC SVM trained applied to WB. (This shows that the prediction of the MED and HIGH risk groups based on training the SVM on the Overlap PBMC gene set is transferrable to the WB dataset. This is the consistency argument we are looking for.)				
**CTRL**	3	0	0	0
**LOW**	3	3	0	2
**MED**	0	0	14	0
**HIGH**	0	0	1	7

We found that the SVM trained on the WB genes were accurate in predicting the medium and high risk SOFA subjects in the PBMC dataset (a), and the overlapping PBMC genes were accurate in predicting the medium and high risk SOFA subjects in the WB dataset (d). Also, training on only the overlapping PBMC genes was sufficient to accurately predict all patients in the PBMC group (c). However, training on all PBMC genes was not useful in predicting in the WB group because the SVM relies heavily on information about genes that were not significant in the WB dataset (b).

## Discussion

In this paper, we show that WB can be used as a surrogate of PBMC expression in a set of critically ill patients who underwent AdHF cardiac surgery. We validate this result with independent set of samples using RT-qPCR. Our results indicate that the direction of change in gene expression profiles of individuals with MOD as compared to controls is similar when determined from PBMC versus WB. In each of the low, medium and high risk SOFA score patients; there was consistency in the direction of gene expression changes. The main enriched terms by GO analysis included those involved in the inflammatory response, apoptosis, and other stress response related pathways. The data revealed that there were 35 significant GO categories and 26 pathways overlapping between PBMC and WB. Additionally, class prediction using machine learning tools demonstrate that the subset of significant genes shared by both PBMC and WB are sufficient to train a predictor separating the SOFA groups.

Overall, multiple explanations can be postulated to support our findings. First, the differences between PBMC and WB could be secondary to lower signal-to-noise ratios in WB, leading to a higher number of false positive rates when tested with multiple comparisons. Thus, a way to reduce the ratio of true positives to true negatives required an additional step in filtering those transcripts with high intraphenotypic variability that would otherwise be removed by multiple hypothesis testing. As a positive consequence, only those transcripts that are more stable across phenotypes are retained for the analysis, providing a more reproducible signal with lower enrichment but higher correlated findings in the PBMC dataset.

Second, while most relevant changes in gene expression are captured by both methods, variability seems larger in the WB leading to many transcripts not being significant when tested with multiple hypotheses.

Third, the globin mRNA depletion method used in the PAXgene stabilized RNA leads to variable changes in gene expression based on different concentrations of WB and content of globin transcripts. Globin reduction protocols [32] **** [34] **** [35] [36] **** [37] have been used to improve microarray data quality by reducing data variability with increased detection rate of expressed genes and improved overlap with the expression signatures of isolated PBMC preparations.

Fourth, the differential composition of cell populations in PBMC and WB might account for differences in GEP patterns. While PBMC seems to directly identify changes in a well-defined immunological set of cells, such an assumption does not apply to WB. In WB, more diverse cell populations including neutrophils, eosinophils, platelets and reticulocytes among other constituents in the preparations, reduce the specific cell population of the study. While they have the potential to be used as biomarkers with the goal of classifying phenotypes, the biological meaning of the results is highly questionable when proposing mechanistic explanations. A comparative analysis revealed that nearly 2,000 genes with at least a 2-fold average difference in expression between WB and PBMCs could reflect the difference in cellular composition [18] **** [22]. Gene expression associated with WB displays a more pronounced pattern of up-regulation **(** **Table 5**). Theoretically, in both PBMC and WB sampling, analysis is possible using microfluidics-based flow cytometry-guided cell sorting followed by subpopulation-specific GEP [38] **** [39]. However, the intention of our approach, in creating a clinically feasible protocol, is to use the integrated mixed PBMC population in the original PBMC protocol and compare it with the WB-approach.

Fifth, the observed differences could also be explained by different RNA processing methods that have an effect on gene expression profile in WB and PBMC samples.

Sixth, the results in RT-qPCR showed non-significant differences between the 10 genes when compared to the microarray results.

Lastly, we have obtained high classification rates using both PBMC and WB. Although PBMC provided more accurate results, classification rates for the moderate and high risk groups were well discriminated by WB which provides significant advantages from a methodological and clinical implementation perspectives.

### Limitations

Our findings should be interpreted in consideration of several study limitations including: 1) limited sample size, 2) lack of power to assess gender specific variation, 3) variation in the time points of collection dictated by clinical setting, 4) use of the SOFA score which has not been specifically developed for the MCS population, and 5) use of cross-sectional single time-point evaluations.

## Conclusions

We conducted a study to evaluate the comparative usefulness of WB and PBMC transcriptome analysis in critically ill patients undergoing a complex and high risk cardiovascular intervention such as MCS therapy. We found that although there was a significant difference in sensitivity, molecular fingerprints of WB and PBMC had a good overlap and concordance in their gene expression with common pathways and mechanisms represented by these genes.

WB as well as PBMC is useful to delineate the inflammatory response associated with MOD after MCS. While PBMC classification outperformed WB, technical refinement and development of larger, prospective studies are warranted to further develop WB biomarkers in critical care settings.

Advantages of PBMC testing include better biological inquiry at the cost of methodological challenges with difficult implementation in the real world clinical setting. While WB potentially carries a reduced value in classification performance and reflection of actual biology, it has the advantage of easier implementation, requires a smaller volume, and is more resistant to operational variation. Even though the critical care setting is associated with many complex interventions, these do not appear to interfere with the ability of using WB biomarkers to identify high risk patients.

## Supporting Information

S1 Table Overlapping up- and down-regulated genes expressed in PBMC and WB between patients after intervention.
(DOCX)

S2 Table Correlations between expression in PBMC and WB, and SOFA score.
(DOCX)

S3 Table GO categories overlapping between PBMC and WB after intervention.
(DOCX)
