# Supplementary material for: Comparison of Whole Blood and Peripheral Blood Mononuclear Cell Gene Expression for Evaluation of the Perioperative Inflammatory Response in Patients with Advanced Heart Failure
Source: PLoS One. 2014 Dec 17;9(12):e115097. doi: 10.1371/journal.pone.0115097 (PMC4269402; doi:10.1371/journal.pone.0115097)
Supplement: S1 Table — Overlapping up- and down-regulated genes expressed in PBMC and WB between patients after intervention. (DOCX) [file pone.0115097.s001.docx]

**Table S1:** Overlapping down and down regulated genes expressed in PBMC and WB between patients after intervention

| **Gene symbol** | **Unique ID** | **p-value** | **Low**  **SOFA score** | | **Medium**  **SOFA score** | | **High**  **SOFA score** | |
| --- | --- | --- | --- | --- | --- | --- | --- | --- |
|  |  |  | **Fold Change (PBMC)** | **Regu-lation** | **Fold Change** | **Regu-lation** | **Fold Change** | **Regu-lation** |
| TBC1D19 |  | 0.050 | -1.098 | down | -1.190 | down | -1.137 | down |
| UBE2F |  | 0.019 | -1.412 | up | 1.791 | up | 1.820 | up |
| BRF2 |  | 0.050 | -1.276 | down | -1.278 | down | -1.132 | down |
| SLC29A2 |  | 0.016 | -1.162 | down | -1.286 | down | -1.403 | down |
| DARC |  | 0.043 | -1.320 | up | 2.191 | up | 3.601 | up |
| MRFAP1L1 |  | 0.005 | -1.535 | down | -1.615 | down | -2.288 | down |
|  | Hs.567480 | 0.031 | -1.210 | down | -1.298 | down | -1.392 | down |
| ZNF589 |  | 0.047 | -1.216 | down | -1.353 | down | -1.415 | down |
| C11orf31 |  | 0.009 | -1.146 | down | -1.233 | down | -1.231 | down |
| LOC92249 |  | 0.006 | -1.421 | down | -1.585 | down | -1.769 | down |
| COPB2 |  | 0.031 | 1.324 | up | 1.450 | up | 1.431 | up |
| ENO2 |  | 0.007 | -1.431 | down | -1.735 | down | -2.222 | down |
| TMEM106B |  | 0.039 | -1.478 | down | -1.557 | down | -1.957 | down |
|  | Hs.551438 | 0.034 | 1.058 | up | 1.184 | up | 1.218 | up |
|  | Hs.497591 | 0.007 | -1.316 | down | -1.411 | down | -1.678 | down |
| WDR45 |  | 0.047 | 1.409 | up | 1.801 | up | 1.833 | up |
| PURA |  | 0.010 | -1.224 | down | -1.395 | down | -1.766 | down |
| LOC728693 |  | 0.014 | -1.197 | down | -1.213 | down | -1.385 | down |
| LOC644988 |  | 0.005 | -1.586 | down | -1.938 | down | -2.336 | down |
| DDHD2 |  | 0.024 | -1.178 | down | -1.186 | down | -1.277 | down |
| CD40LG |  | 0.005 | -2.066 | down | -2.702 | down | -3.653 | down |
| CD40LG |  | 0.005 | -2.066 | down | -2.702 | down | -3.653 | down |
| FAM84B |  | 0.006 | -1.939 | down | -2.182 | down | -2.584 | down |
| C21orf51 |  | 0.047 | -1.168 | down | -1.243 | down | -1.447 | down |
| NCALD |  | 0.005 | -1.783 | down | -2.016 | down | -3.357 | down |
| NCALD |  | 0.005 | -1.783 | down | -2.016 | down | -3.357 | down |
| CTSB |  | 0.005 | 1.497 | up | 2.045 | up | 2.669 | up |
| C16orf79 |  | 0.024 | -1.314 | down | -1.459 | down | -1.288 | down |
| LOC441743 |  | 0.009 | -1.474 | down | -1.925 | down | -2.245 | down |
| CD8B |  | 0.006 | -1.563 | down | -1.798 | down | -2.359 | down |
| LCK |  | 0.005 | -1.848 | down | -2.431 | down | -3.232 | down |
| FCGBP |  | 0.005 | -2.514 | down | -2.900 | down | -4.858 | down |
| LBA1 |  | 0.011 | -1.214 | down | -1.507 | down | -1.979 | down |
| SORT1 |  | 0.005 | 2.028 | up | 2.189 | up | 2.718 | up |
| C16orf30 |  | 0.005 | -1.857 | down | -2.487 | down | -3.952 | down |
| TCF7 |  | 0.007 | -1.610 | down | -1.873 | down | -2.248 | down |
| MRI1 |  | 0.019 | -1.472 | down | -1.699 | down | -1.779 | down |
| APRT |  | 0.009 | -1.196 | down | -1.621 | down | -1.556 | down |
| ETS1 |  | 0.005 | -1.651 | down | -2.482 | down | -3.832 | down |
| TCF7 |  | 0.006 | -1.857 | down | -2.107 | down | -2.504 | down |
| TRAT1 |  | 0.005 | -1.674 | down | -2.339 | down | -4.325 | down |
| CLIC4 |  | 0.005 | -1.701 | down | -2.240 | down | -2.713 | down |
| IL2RB |  | 0.005 | -2.473 | down | -2.879 | down | -8.016 | down |
| SAMD3 |  | 0.005 | -2.139 | down | -2.435 | down | -4.728 | down |
| AMPH |  | 0.005 | 1.471 | up | 2.048 | up | 3.681 | up |
| MAGED1 |  | 0.034 | -1.174 | down | -1.272 | down | -1.767 | down |
| LAT |  | 0.007 | -1.763 | down | -2.172 | down | -2.760 | down |
| LOC653079 |  | 0.037 | -1.190 | down | -1.216 | down | -1.686 | down |
|  | Hs.276860 | 0.006 | -1.612 | down | -1.752 | down | -2.748 | down |
|  | Hs.554324 | 0.005 | -2.443 | down | -4.067 | down | -11.282 | down |
| LAX1 |  | 0.006 | -1.581 | down | -1.970 | down | -2.995 | down |
| LILRA5 |  | 0.007 | 1.885 | up | 2.842 | up | 2.648 | up |
| CCL5 |  | 0.005 | -1.559 | down | -1.975 | down | -6.794 | down |
| BIN1 |  | 0.005 | -1.821 | down | -2.543 | down | -3.608 | down |
| GADD45A |  | 0.014 | 1.801 | up | 2.012 | up | 3.178 | up |
| CD8A |  | 0.005 | -2.182 | down | -3.299 | down | -8.575 | down |
| ICAM2 |  | 0.018 | -1.637 | down | -1.606 | down | -2.065 | down |
| CD79B |  | 0.005 | -1.642 | down | -1.536 | down | -3.059 | down |
| PHF15 |  | 0.011 | -1.631 | down | -1.735 | down | -2.383 | down |
| LYSMD2 |  | 0.024 | -1.452 | down | -1.412 | down | -2.385 | down |
| KANK3 |  | 0.048 | -1.164 | down | -1.238 | down | -1.198 | down |
| HLA-DQA1 |  | 0.009 | -1.778 | down | -2.170 | down | -6.841 | down |
| FLT3LG |  | 0.005 | -1.801 | down | -2.690 | down | -4.592 | down |
| PSME2 |  | 0.048 | -1.024 | down | 1.122 | up | -1.607 | down |
| FYN |  | 0.009 | -1.375 | down | -1.581 | down | -1.851 | down |
| SAMD3 |  | 0.005 | -1.970 | down | -2.352 | down | -5.068 | down |
| BCL11B |  | 0.005 | -2.283 | down | -3.478 | down | -8.911 | down |
| PLCG1 |  | 0.010 | -1.892 | down | -2.205 | down | -2.522 | down |
| LTA |  | 0.005 | -1.501 | down | -1.749 | down | -2.653 | down |
| KIAA1147 |  | 0.005 | -1.590 | down | -2.233 | down | -3.050 | down |
| C19orf59 |  | 0.005 | 2.843 | up | 5.235 | up | 7.157 | up |
| CD3G |  | 0.005 | -1.719 | down | -2.177 | down | -3.780 | down |
| CD3D |  | 0.005 | -2.027 | down | -3.295 | down | -7.713 | down |
| HLA-DPA1 |  | 0.009 | -1.367 | down | -1.461 | down | -3.783 | down |
| TXK |  | 0.006 | -1.631 | down | -2.003 | down | -2.597 | down |
| ZMYND11 |  | 0.007 | -1.624 | down | -2.017 | down | -2.457 | down |
| PHCA |  | 0.010 | 2.052 | up | 2.199 | up | 2.577 | up |
| ATL3 |  | 0.006 | -1.434 | down | 1.676 | up | 2.501 | up |
| CD27 |  | 0.006 | -1.775 | down | -2.822 | down | -5.611 | down |
| GZMK |  | 0.005 | -3.674 | down | -5.039 | down | -9.928 | down |
| ARL4C |  | 0.005 | -1.478 | down | -1.953 | down | -2.594 | down |
| CUEDC1 |  | 0.046 | -1.528 | down | 1.521 | up | 1.795 | up |
| ZNF827 |  | 0.005 | -1.461 | down | -1.888 | down | -2.124 | down |
| LDHB |  | 0.005 | -1.765 | down | -2.525 | down | -3.106 | down |
| IL32 |  | 0.005 | -1.569 | down | -2.172 | down | -3.799 | down |
| JAM3 |  | 0.018 | 1.609 | up | 1.250 | up | -1.747 | down |
| S100A12 |  | 0.024 | 2.435 | up | 2.663 | up | 3.324 | up |
| RAB3IP |  | 0.017 | -1.827 | down | -2.071 | down | -2.058 | down |
| VSIG4 |  | 0.005 | 1.257 | up | 1.962 | up | 10.599 | up |
| PTPRCAP |  | 0.005 | -1.841 | down | -2.946 | down | -6.135 | down |
| KLF12 |  | 0.005 | -1.929 | down | -2.335 | down | -3.646 | down |
| LOC644511 |  | 0.006 | -1.422 | down | -1.754 | down | -2.093 | down |
| RUNX3 |  | 0.005 | -1.826 | down | -2.068 | down | -3.196 | down |
| S1PR5 |  | 0.005 | -2.610 | down | -2.857 | down | -7.143 | down |
| LOC202134 |  | 0.005 | -1.999 | down | -2.658 | down | -3.355 | down |
|  | Hs.436134 | 0.017 | -1.187 | down | -1.602 | down | -1.996 | down |
| CARD11 |  | 0.011 | -1.693 | down | -1.880 | down | -3.588 | down |
| CLSTN1 |  | 0.008 | -1.328 | down | -1.525 | down | -1.911 | down |
| DENND2D |  | 0.005 | -1.797 | down | -2.202 | down | -3.487 | down |
| LOC644132 |  | 0.005 | 1.542 | up | 1.514 | up | 2.421 | up |
| CCND2 |  | 0.016 | -1.397 | down | -1.800 | down | -2.323 | down |
| CD3D |  | 0.005 | -2.026 | down | -3.154 | down | -7.734 | down |
| ITM2A |  | 0.005 | -1.913 | down | -2.792 | down | -5.529 | down |
| CCDC102A |  | 0.008 | -1.639 | down | -1.733 | down | -2.893 | down |
| CA5B |  | 0.026 | -1.717 | down | -1.994 | down | -2.066 | down |
| RPL3 |  | 0.007 | -1.419 | down | -1.716 | down | -2.185 | down |
| ST6GAL1 |  | 0.005 | -1.332 | down | -1.632 | down | -2.877 | down |
| DOCK10 |  | 0.006 | -1.375 | down | -1.632 | down | -2.339 | down |
| IL1R2 |  | 0.011 | 1.135 | up | 1.976 | up | 15.480 | up |
| KLRD1 |  | 0.005 | -1.941 | down | -2.374 | down | -6.028 | down |
| CD79A |  | 0.005 | -1.842 | down | -1.809 | down | -3.061 | down |
| CD3E |  | 0.005 | -1.837 | down | -2.734 | down | -4.568 | down |
| PTGDS |  | 0.009 | -1.752 | down | -2.099 | down | -3.329 | down |
| TLR9 |  | 0.045 | -1.168 | down | -1.240 | down | -1.277 | down |
| GZMB |  | 0.006 | -2.316 | down | -2.226 | down | -7.583 | down |
| FAM102A |  | 0.005 | -1.758 | down | -2.375 | down | -3.128 | down |
| LOC387841 |  | 0.005 | -1.759 | down | -2.614 | down | -4.960 | down |
| ZNF831 |  | 0.005 | -1.580 | down | -1.858 | down | -2.198 | down |
| LOC728734 |  | 0.021 | -1.217 | down | -1.557 | down | -1.585 | down |
| P2RY10 |  | 0.005 | -1.775 | down | -2.062 | down | -2.925 | down |
| GBP4 |  | 0.021 | -1.393 | down | -1.277 | down | -3.159 | down |
| FBXO32 |  | 0.011 | -1.219 | down | -1.514 | down | -1.939 | down |
| KIAA0907 |  | 0.012 | -1.529 | down | -1.743 | down | -2.090 | down |
| CX3CR1 |  | 0.007 | -1.200 | down | -1.012 | down | -3.954 | down |
| PAQR8 |  | 0.018 | -1.323 | down | -1.616 | down | -1.752 | down |
| PASK |  | 0.009 | -2.407 | down | -2.563 | down | -3.232 | down |
| CD160 |  | 0.005 | -2.092 | down | -2.777 | down | -5.310 | down |
| PLCG1 |  | 0.005 | -1.872 | down | -2.780 | down | -4.013 | down |
| LOC392437 |  | 0.005 | 1.491 | up | 1.720 | up | 2.048 | up |
| LPAR5 |  | 0.007 | -1.481 | down | -1.617 | down | -2.641 | down |
| CD320 |  | 0.012 | -1.156 | down | -1.332 | down | -2.107 | down |
| MAP4 |  | 0.044 | 1.080 | up | 1.080 | up | 1.233 | up |
| LOC100133372 |  | 0.047 | -1.542 | down | -1.458 | down | -1.903 | down |
| LPXN |  | 0.021 | -1.228 | down | -1.264 | down | -1.817 | down |
| AHSP |  | 0.014 | 23.029 | up | 28.623 | up | 64.306 | up |
| IRAK3 |  | 0.005 | -1.959 | down | 2.441 | up | 3.334 | up |
| LRRN3 |  | 0.009 | -1.860 | down | -3.023 | down | -3.639 | down |
| PLEKHF1 |  | 0.010 | -2.244 | down | -2.288 | down | -4.410 | down |
| CD63 |  | 0.005 | 1.820 | up | 2.202 | up | 3.634 | up |
| FBXO21 |  | 0.008 | -1.387 | down | -1.476 | down | -1.827 | down |
| ABLIM1 |  | 0.005 | -1.793 | down | -2.969 | down | -5.737 | down |
| SH3YL1 |  | 0.011 | -1.560 | down | -2.106 | down | -2.547 | down |
| C19orf2 |  | 0.016 | -1.469 | down | -1.476 | down | -1.823 | down |
| FKBP5 |  | 0.005 | 1.572 | up | 1.960 | up | 3.196 | up |
| PRAGMIN |  | 0.005 | -1.874 | down | -2.450 | down | -4.114 | down |
| C19orf60 |  | 0.011 | -1.559 | down | -1.773 | down | -1.975 | down |
| RARRES3 |  | 0.005 | -1.750 | down | -2.000 | down | -5.157 | down |
| TRADD |  | 0.006 | -1.453 | down | -1.795 | down | -2.209 | down |
| SIRPG |  | 0.006 | -1.499 | down | -1.776 | down | -2.396 | down |
| LOC652071 |  | 0.013 | -1.261 | down | -1.404 | down | -1.605 | down |
| MGC3020 |  | 0.006 | -1.434 | down | -1.608 | down | -1.971 | down |
| CD96 |  | 0.005 | -2.256 | down | -2.748 | down | -4.935 | down |
|  | Hs.534439 | 0.005 | -2.315 | down | -2.774 | down | -5.863 | down |
| IL11RA |  | 0.006 | -1.783 | down | -2.298 | down | -3.334 | down |
| RASGRP1 |  | 0.005 | -2.197 | down | -3.352 | down | -6.931 | down |
| BTLA |  | 0.010 | -1.181 | down | -1.350 | down | -1.948 | down |
| KLHL3 |  | 0.005 | -1.669 | down | -2.075 | down | -2.741 | down |
| LEF1 |  | 0.005 | -1.890 | down | -3.584 | down | -7.212 | down |
| GRB10 |  | 0.005 | 1.425 | up | 1.632 | up | 2.538 | up |
| QSOX1 |  | 0.005 | 1.681 | up | 1.921 | up | 2.970 | up |
| TMEM204 |  | 0.005 | -1.941 | down | -2.638 | down | -4.120 | down |
| CD8A |  | 0.007 | -2.309 | down | -2.735 | down | -4.608 | down |
| SP140 |  | 0.009 | -1.258 | down | -1.348 | down | -1.987 | down |
| CROP |  | 0.006 | -1.494 | down | -2.065 | down | -2.383 | down |
| LOC100131196 |  | 0.014 | -1.189 | down | -1.501 | down | -1.649 | down |
| GIMAP7 |  | 0.019 | -1.750 | down | -1.896 | down | -3.499 | down |
| CD8A |  | 0.006 | -2.283 | down | -3.234 | down | -8.740 | down |
| ETF1 |  | 0.011 | 1.234 | up | 1.252 | up | 1.443 | up |
| LY9 |  | 0.005 | -1.875 | down | -2.280 | down | -4.370 | down |
| LOC286208 |  | 0.007 | -1.289 | down | -1.628 | down | -1.863 | down |
| BCL2 |  | 0.005 | -1.341 | down | -1.854 | down | -3.008 | down |
| SERPINB1 |  | 0.006 | 1.723 | up | 1.861 | up | 2.981 | up |
| TGFBR3 |  | 0.005 | -2.321 | down | -3.014 | down | -6.259 | down |
| PRKCH |  | 0.005 | -2.019 | down | -2.766 | down | -5.184 | down |
| FCER1A |  | 0.005 | -2.269 | down | -3.763 | down | -10.177 | down |
| MVP |  | 0.041 | 1.203 | up | 1.340 | up | 1.421 | up |
| DKFZp761P0423 |  | 0.005 | -1.629 | down | -2.022 | down | -3.019 | down |
| SERINC2 |  | 0.005 | 1.298 | up | 1.563 | up | 2.334 | up |
| RECK |  | 0.005 | -1.487 | down | -1.715 | down | -2.098 | down |
| KLRD1 |  | 0.005 | -1.963 | down | -2.455 | down | -5.449 | down |
| IGFBP7 |  | 0.027 | 1.379 | up | 1.620 | up | 2.172 | up |
| GZMA |  | 0.006 | -2.062 | down | -2.194 | down | -7.656 | down |
| IL32 |  | 0.006 | -1.934 | down | -3.032 | down | -6.146 | down |
| TRAF3IP3 |  | 0.005 | -1.888 | down | -2.035 | down | -3.763 | down |
| CCND2 |  | 0.007 | -1.515 | down | -1.893 | down | -2.738 | down |
| LOC440311 |  | 0.010 | -2.538 | down | -3.409 | down | -3.601 | down |
| GLTSCR2 |  | 0.016 | -1.261 | down | -1.405 | down | -1.659 | down |
| LAT |  | 0.020 | -1.447 | down | -1.620 | down | -1.912 | down |
| LOC653881 |  | 0.008 | -1.248 | down | -1.523 | down | -1.722 | down |
| FLOT1 |  | 0.008 | 1.451 | up | 1.683 | up | 2.101 | up |
| NCR3 |  | 0.006 | -1.776 | down | -2.085 | down | -2.664 | down |
| SLC2A3 |  | 0.027 | 1.530 | up | 1.690 | up | 1.995 | up |
| UBASH3A |  | 0.005 | -2.327 | down | -3.110 | down | -4.911 | down |
| IL7R |  | 0.005 | -2.319 | down | -3.891 | down | -7.479 | down |
| KIAA0748 |  | 0.005 | -1.567 | down | -2.037 | down | -2.423 | down |
| EFHC2 |  | 0.026 | -1.112 | down | -1.284 | down | -1.371 | down |
| FAM102A |  | 0.005 | -2.212 | down | -2.908 | down | -4.824 | down |
| LRFN3 |  | 0.005 | -1.556 | down | -1.921 | down | -2.930 | down |
| LOC728782 |  | 0.006 | -1.414 | down | -1.614 | down | -2.142 | down |
| SPOCK2 |  | 0.005 | -1.935 | down | -3.351 | down | -5.789 | down |
| LOC653316 |  | 0.008 | -1.466 | down | -1.694 | down | -1.841 | down |
| SBK1 |  | 0.005 | -2.070 | down | -2.698 | down | -5.390 | down |
| CXCR5 |  | 0.010 | -1.858 | down | -2.112 | down | -2.693 | down |
| IL7R |  | 0.005 | -2.418 | down | -3.905 | down | -8.217 | down |
| CD247 |  | 0.005 | -2.436 | down | -3.467 | down | -9.013 | down |
| LOC728014 |  | 0.005 | -1.916 | down | -2.114 | down | -3.303 | down |
| NMT2 |  | 0.005 | -2.280 | down | -2.973 | down | -4.877 | down |
|  | Hs.567464 | 0.005 | -2.436 | down | -2.936 | down | -4.608 | down |
| PLEKHA1 |  | 0.005 | -1.547 | down | -1.999 | down | -2.759 | down |
| SIRPG |  | 0.007 | -1.491 | down | -1.670 | down | 2.321 | down |
| OR7E156P |  | 0.027 | -1.333 | down | -1.328 | down | -1.343 | down |
| NCF1 |  | 0.037 | 1.522 | up | 1.928 | up | -1.029 | down |
| TNFRSF25 |  | 0.005 | -2.686 | down | -4.596 | down | -8.353 | down |
| ITGB7 |  | 0.005 | -1.544 | down | -2.042 | down | -3.023 | down |
| TCP11L2 |  | 0.033 | 1.386 | up | 1.617 | up | 2.475 | up |
| RPL10A |  | 0.010 | -1.202 | down | -1.622 | down | -1.939 | down |
| C6orf190 |  | 0.005 | -1.765 | down | -2.202 | down | -3.744 | down |
| TCEA3 |  | 0.009 | -1.588 | down | -2.129 | down | -3.834 | down |
| GPR18 |  | 0.005 | -2.012 | down | -2.019 | down | -3.331 | down |
| CTSL1 |  | 0.008 | 1.399 | up | 2.362 | up | 4.837 | up |
| BCL2 |  | 0.005 | -1.412 | down | -2.056 | down | -3.868 | down |
| HLA-DOA |  | 0.005 | -1.351 | down | -1.793 | down | -2.302 | down |
| FAM113B |  | 0.005 | -1.538 | down | -2.243 | down | -5.100 | down |
| HLA-DRB6 |  | 0.009 | -1.113 | down | -1.385 | down | -2.529 | down |
| CD2 |  | 0.005 | -2.245 | down | -3.349 | down | -7.121 | down |
| SKAP1 |  | 0.005 | -2.114 | down | -2.809 | down | -5.954 | down |
| KLRF1 |  | 0.005 | -2.180 | down | -3.199 | down | -4.835 | down |
| AKR1B1 |  | 0.030 | -1.257 | down | -1.428 | down | -1.577 | down |
| LOC100131609 |  | 0.040 | -1.404 | down | -1.404 | down | -1.580 | down |
| TMC6 |  | 0.016 | -1.571 | down | -1.798 | down | -1.941 | down |
| 1-Sep |  | 0.005 | -1.785 | down | -2.147 | down | -3.201 | down |
| EBI2 |  | 0.008 | -2.529 | down | -3.508 | down | -4.332 | down |
| C5orf32 |  | 0.005 | 1.896 | up | 2.462 | up | 4.724 | up |
| LEF1 |  | 0.005 | -1.923 | down | -3.338 | down | -7.793 | down |
| PRF1 |  | 0.005 | -2.082 | down | -2.361 | down | -6.594 | down |
| FBLN5 |  | 0.024 | -1.408 | down | -1.574 | down | -1.756 | down |
| CDC25B |  | 0.005 | -1.827 | down | -2.571 | down | -3.387 | down |
| LOC136143 |  | 0.026 | -1.644 | down | -2.096 | down | -1.923 | down |
| PTCRA |  | 0.036 | 1.336 | up | 1.238 | up | 1.060 | up |
| TMEM170B |  | 0.032 | 1.790 | up | 1.838 | up | 2.259 | up |
| ZAP70 |  | 0.005 | -1.827 | down | -2.576 | down | -3.897 | down |
| IL12RB1 |  | 0.018 | -1.365 | down | -1.460 | down | -1.859 | down |
| PAOX |  | 0.042 | -1.225 | down | -1.218 | down | -1.756 | down |
| TAGAP |  | 0.015 | -1.264 | down | -1.472 | down | -1.731 | down |
| INPP4B |  | 0.005 | -1.335 | down | -1.771 | down | -2.382 | down |
| GADD45A |  | 0.010 | 1.849 | up | 2.622 | up | 3.929 | up |
| GPR56 |  | 0.006 | -2.228 | down | -2.612 | down | -9.470 | down |
| GSTO1 |  | 0.008 | 1.426 | up | 1.442 | up | 1.825 | up |
| HAPLN3 |  | 0.009 | -1.400 | down | -1.633 | down | -2.095 | down |
| RPL21 |  | 0.021 | -1.145 | down | -1.266 | down | -1.590 | down |
| SH3KBP1 |  | 0.008 | -1.566 | down | -1.716 | down | -2.650 | down |
| GNLY |  | 0.005 | -1.971 | down | -2.134 | down | -3.989 | down |
| RLTPR |  | 0.005 | -1.432 | down | -1.664 | down | -2.012 | down |
| CD5 |  | 0.005 | -1.762 | down | -2.571 | down | -3.593 | down |
| PLD1 |  | 0.005 | 1.277 | up | 1.471 | up | 1.845 | up |
| BIN1 |  | 0.005 | -2.004 | down | -2.368 | down | -3.856 | down |
| HLA-F |  | 0.005 | -1.827 | down | -2.253 | down | -3.541 | down |
| ECHDC3 |  | 0.025 | 1.217 | up | 1.271 | up | 2.006 | up |
| ATG7 |  | 0.017 | 1.701 | up | 1.809 | up | 2.101 | up |
| GRAP |  | 0.005 | -1.879 | down | -2.474 | down | -4.118 | down |
| ARG1 |  | 0.010 | 2.559 | up | 2.303 | up | 5.996 | up |
| LTB |  | 0.008 | -1.638 | down | -2.255 | down | -5.178 | down |
| LOC100133678 |  | 0.009 | -1.681 | down | -2.135 | down | -7.098 | down |
| GNLY |  | 0.005 | -2.189 | down | -2.584 | down | -9.530 | down |
| GIMAP5 |  | 0.010 | -1.728 | down | -2.179 | down | -3.554 | down |
| TAGAP |  | 0.017 | -1.513 | down | -1.704 | down | -2.029 | down |
| CCR7 |  | 0.006 | -2.028 | down | -3.642 | down | -8.011 | down |
| PIK3IP1 |  | 0.007 | -2.300 | down | -2.804 | down | -4.214 | down |
| LTB |  | 0.005 | -2.271 | down | -3.488 | down | -7.558 | down |
| MCOLN2 |  | 0.005 | -1.445 | down | -1.869 | down | -3.002 | down |
| PPP1R16B |  | 0.005 | -1.497 | down | -1.927 | down | -2.885 | down |
| SLC30A1 |  | 0.010 | 1.462 | up | 1.524 | up | 2.019 | up |
| GPR56 |  | 0.007 | -2.177 | down | -2.639 | down | -8.150 | down |
| CX3CR1 |  | 0.011 | -1.139 | down | -1.061 | down | -2.987 | down |
| LCK |  | 0.005 | -1.572 | down | -2.150 | down | -2.568 | down |
| MATK |  | 0.019 | -1.999 | down | -2.153 | down | -4.255 | down |
| GOLGA8B |  | 0.005 | -1.942 | down | -3.196 | down | -4.573 | down |
| ANO9 |  | 0.005 | -1.452 | down | -1.670 | down | -1.931 | down |
| LOC387882 |  | 0.013 | -1.067 | down | -1.181 | down | -3.563 | down |
| SIGIRR |  | 0.007 | -1.854 | down | -1.941 | down | -2.832 | down |
| SKAP1 |  | 0.005 | -1.798 | down | -2.269 | down | -3.537 | down |
| SH2D1A |  | 0.005 | -2.280 | down | -3.077 | down | -5.642 | down |
| RPS4X |  | 0.014 | -1.296 | down | -1.790 | down | -1.804 | down |
| ARHGEF18 |  | 0.024 | -1.570 | down | -1.590 | down | -1.979 | down |
| ITPR3 |  | 0.005 | -1.635 | down | -2.049 | down | -3.442 | down |
|  | Hs.572649 | 0.005 | -2.488 | down | -3.251 | down | -4.457 | down |
| CD247 |  | 0.005 | -2.471 | down | -3.311 | down | -9.076 | down |
| CD7 |  | 0.005 | -2.018 | down | -2.986 | down | -5.528 | down |
| DYRK2 |  | 0.005 | -1.809 | down | -2.144 | down | -3.093 | down |
| ETS1 |  | 0.005 | -1.984 | down | -3.320 | down | -5.182 | down |
| SLC7A6 |  | 0.006 | -1.383 | down | -1.602 | down | -1.955 | down |
| CA4 |  | 0.014 | 1.530 | up | 1.532 | up | 1.978 | up |
| ABLIM1 |  | 0.005 | -1.940 | down | -2.520 | down | -4.952 | down |
|  | Hs.560343 | 0.005 | -1.669 | down | -2.094 | down | -3.161 | down |
| PVRIG |  | 0.005 | -2.406 | down | -3.132 | down | -6.459 | down |
| OCIAD2 |  | 0.005 | -1.964 | down | -2.559 | down | -4.544 | down |
| LIME1 |  | 0.005 | -1.668 | down | -2.378 | down | -4.707 | down |
| CD79B |  | 0.005 | -1.734 | down | -1.600 | down | -3.244 | down |
| ESYT1 |  | 0.005 | -1.382 | down | -1.621 | down | -2.024 | down |
| MYC |  | 0.022 | -1.383 | down | -1.770 | down | -2.474 | down |
| EVL |  | 0.005 | -2.966 | down | -3.335 | down | -7.267 | down |
| GNLY |  | 0.005 | -2.302 | down | -2.738 | down | -9.103 | down |
| CTSW |  | 0.017 | -1.551 | down | -1.758 | down | -2.999 | down |
| HP |  | 0.011 | -3.279 | down | 4.755 | up | 6.795 | up |
| CD6 |  | 0.005 | -2.161 | down | -3.312 | down | -7.367 | down |
| PLEKHA1 |  | 0.005 | -2.201 | down | -2.738 | down | -4.430 | down |
| MYL6 |  | 0.006 | -1.514 | down | 1.802 | up | 1.820 | up |
|  | Hs.546375 | 0.005 | -2.457 | down | -3.380 | down | -8.927 | down |
| HNRPDL |  | 0.023 | -1.629 | down | -1.696 | down | -1.907 | down |
| TSPO |  | 0.008 | -1.659 | down | 1.794 | up | 2.159 | up |
| LOC728481 |  | 0.038 | -1.340 | down | -1.923 | down | -2.181 | down |
| ATP8B2 |  | 0.005 | -1.758 | down | -2.300 | down | -4.840 | down |
| EPHX2 |  | 0.005 | -1.891 | down | -2.204 | down | -3.237 | down |
| PYHIN1 |  | 0.005 | -2.257 | down | -2.716 | down | -5.466 | down |
| RAB22A |  | 0.018 | -1.297 | down | -1.244 | down | -1.788 | down |
| LRRFIP2 |  | 0.005 | -1.383 | down | 1.632 | up | 1.832 | up |
|  | Hs.443123 | 0.016 | -1.219 | down | -1.417 | down | -1.436 | down |
| USP36 |  | 0.010 | -1.478 | down | -1.658 | down | -2.335 | down |
| SLAMF6 |  | 0.006 | -1.935 | down | -2.348 | down | -5.105 | down |
| PDCD7 |  | 0.032 | -1.384 | down | -1.601 | down | -1.698 | down |
| STAT4 |  | 0.005 | -2.161 | down | -3.066 | down | -5.837 | down |
| EOMES |  | 0.007 | -2.234 | down | -2.683 | down | -5.263 | down |
| C5orf39 |  | 0.005 | -1.942 | down | -2.604 | down | -3.911 | down |
| PYHIN1 |  | 0.005 | -2.212 | down | -2.466 | down | -4.456 | down |
| LOC644237 |  | 0.007 | -1.676 | down | 1.511 | up | 2.295 | up |
| ITK |  | 0.005 | -1.872 | down | -2.827 | down | -5.658 | down |
| CCL5 |  | 0.011 | -1.232 | down | -1.266 | down | -4.357 | down |
| TBC1D19 |  | 0.050 | -1.098 | down | -1.190 | down | -1.137 | down |
| UBE2F |  | 0.019 | -1.412 | down | 1.791 | up | 1.820 | up |
| BRF2 |  | 0.050 | -1.276 | down | -1.278 | down | -1.132 | down |
| SLC29A2 |  | 0.016 | -1.162 | down | -1.286 | down | -1.403 | down |
| DARC |  | 0.043 | -1.320 | down | 2.191 | up | 3.601 | up |
| MRFAP1L1 |  | 0.005 | -1.535 | down | -1.615 | down | -2.288 | down |
