# Supplementary material for: Comparison of Whole Blood and Peripheral Blood Mononuclear Cell Gene Expression for Evaluation of the Perioperative Inflammatory Response in Patients with Advanced Heart Failure
Source: PLoS One. 2014 Dec 17;9(12):e115097. doi: 10.1371/journal.pone.0115097 (PMC4269402; doi:10.1371/journal.pone.0115097)
Supplement: S2 Table — Correlations between expression in PBMC and WB, and SOFA score. (DOCX) [file pone.0115097.s002.docx]

**Table S2:**  Correlations between expression in PBMC and WB, and SOFA score.

| **ProbeID** | **Gene Symbol** | **Cor(PBMC, WB)** | **Cor(WB, SOFA)** | **Cor(PBMC, SOFA)** |
| --- | --- | --- | --- | --- |
| 650553 | BIN1 | 0.845281651 | -0.836261155 | -0.63315578 |
| 4670441 | FBLN5 | 0.734725453 | -0.536217092 | -0.504547971 |
| 7320370 | STAT4 | 0.925107518 | -0.703744032 | -0.710558676 |
| 4830301 | KAT2A | 0.690180002 | -0.671122213 | -0.629244108 |
| 770411 | PHF15 | 0.743416164 | -0.830468654 | -0.569209833 |
| 7550592 | LOC644237 | 0.762499504 | 0.672972922 | 0.621510544 |
| 7330097 | ADARB1 | 0.828531769 | -0.633413105 | -0.443903767 |
| 5670605 | MATK | 0.878256461 | -0.685590239 | -0.606167543 |
| 620136 | LILRA5 | 0.60669278 | 0.622409893 | 0.412080864 |
| 4640576 | PRKCQ | 0.918842642 | -0.765464607 | -0.72412132 |
| 1940296 | LOC387841 | 0.922298563 | -0.719314392 | -0.715882548 |
| 160746 | APRT | 0.609628238 | -0.721832566 | -0.455304419 |
| 770167 | CD79B | 0.820538094 | -0.720523237 | -0.699505767 |
| 5860373 | SIGIRR | 0.7754228 | -0.719941697 | -0.647821293 |
| 7570195 | KIAA0355 | 0.755960882 | -0.472976929 | -0.601651796 |
| 130609 | FCGBP | 0.919814411 | -0.593161516 | -0.639178282 |
| 5270132 | CBLB | 0.790842572 | -0.639787093 | -0.632288524 |
| 7320372 | EOMES | 0.905891425 | -0.681162968 | -0.685350092 |
| 3990379 | ITGB7 | 0.842661797 | -0.753490402 | -0.642031738 |
| 6580041 | GNLY | 0.924916545 | -0.748607221 | -0.707785548 |
| 6400564 |  | 0.80784255 | -0.632083113 | -0.721290539 |
| 4810333 | IL12RB1 | 0.774209599 | -0.707522505 | -0.477809282 |
| 580102 |  | 0.924173027 | -0.770481857 | -0.686706655 |
| 6580408 | CTSW | 0.865139219 | -0.63056706 | -0.568218883 |
| 3140709 | LOC100131196 | 0.556839776 | -0.576420855 | -0.499656406 |
| 2690020 | LOC652071 | 0.602236631 | -0.692198944 | -0.645888517 |
| 4860050 | FYN | 0.758618299 | -0.665335197 | -0.688758387 |
| 3180494 | BCL2 | 0.867809417 | -0.697068515 | -0.716266552 |
| 4150201 | BCL2 | 0.925331253 | -0.703162706 | -0.765974015 |
| 510762 | FAM62B | 0.76769888 | -0.452739699 | -0.261656505 |
| 2600040 | C19orf2 | 0.602380355 | -0.63057709 | -0.47255544 |
| 4860762 | INPP4B | 0.693721642 | -0.662012783 | -0.685066198 |
| 7570324 | ID3 | 0.666844238 | -0.600173205 | -0.54732338 |
| 70286 | LOC441743 | 0.372423909 | -0.527271199 | -0.592487071 |
| 5870008 |  | 0.953284895 | -0.782777893 | -0.680325403 |
| 4920612 | GNLY | 0.878102763 | -0.549253906 | -0.584701943 |
| 5360064 | GNLY | 0.960721541 | -0.726791087 | -0.675916025 |
| 4220053 | SKAP1 | 0.908645484 | -0.790024192 | -0.681115428 |
| 1450504 |  | 0.586936291 | -0.664577971 | -0.564802399 |
| 3190112 | SERPINB1 | 0.858311603 | 0.772479262 | 0.707285995 |
| 2690189 | MGC3020 | 0.765541322 | -0.604089831 | -0.582583406 |
| 4280692 | PARP1 | 0.711375784 | -0.689014856 | -0.616030586 |
| 6900048 | LOC648210 | 0.164790598 | -0.577882645 | -0.425160621 |
| 6400243 | ABLIM1 | 0.885229604 | -0.688486476 | -0.816313556 |
| 1030296 | BCL11B | 0.890974059 | -0.72777134 | -0.758389999 |
| 4760431 | LOC136143 | 0.572293696 | -0.662510242 | -0.321615286 |
| 1030167 | SAMD3 | 0.855671795 | -0.624629676 | -0.721813038 |
| 1710369 | RPL3 | 0.627840568 | -0.620141512 | -0.632334473 |
| 2680050 | SIRPG | 0.743622208 | -0.743469554 | -0.719254745 |
| 4900070 | GSTO1 | 0.885666117 | 0.736253759 | 0.711851242 |
| 1430341 | PTPRCAP | 0.874090255 | -0.774164625 | -0.718808007 |
| 2760239 | RASGRP1 | 0.842481283 | -0.695710722 | -0.804642539 |
| 3830220 | UBASH3A | 0.929920179 | -0.707516114 | -0.677528507 |
| 1740451 | IL1R2 | 0.908143491 | 0.770533913 | 0.73787952 |
| 4570255 | LEF1 | 0.906518564 | -0.719769069 | -0.724753299 |
| 2810601 | LEF1 | 0.946417631 | -0.733063168 | -0.707600694 |
| 7560632 | ITK | 0.878780472 | -0.733399372 | -0.726457078 |
| 290402 | IL2RB | 0.880347067 | -0.73331674 | -0.765448993 |
| 2570112 | ABLIM1 | 0.929223459 | -0.849095675 | -0.770935617 |
| 4050491 | TCEA3 | 0.824683066 | -0.633568475 | -0.690867171 |
| 3830349 | IL7R | 0.898457136 | -0.736999608 | -0.654049475 |
| 1440564 | RUNX3 | 0.870917044 | -0.734129337 | -0.674831944 |
| 2570288 | SH3YL1 | 0.76541928 | -0.571206531 | -0.576722483 |
| 1430711 | KLF12 | 0.769609087 | -0.645598391 | -0.74252293 |
| 2070168 | CX3CR1 | 0.85412975 | -0.7001199 | -0.711073046 |
| 5550373 | CX3CR1 | 0.80963988 | -0.669825069 | -0.75975637 |
| 4780504 | RNU6-15 | 0.764877418 | 0.729069621 | 0.45462982 |
| 3610286 | GLTSCR2 | 0.697265126 | -0.721565198 | -0.624043296 |
| 1400240 | LDHB | 0.799462654 | -0.655721908 | -0.587372099 |
| 1240243 | ATL3 | 0.898893256 | 0.721067404 | 0.713120275 |
| 4670193 | PRF1 | 0.936155968 | -0.685169167 | -0.67759677 |
| 3190379 | TGFBR3 | 0.933487468 | -0.672571086 | -0.670715164 |
| 6330553 | DYRK2 | 0.774723029 | -0.740224643 | -0.75992805 |
| 7050136 | RAB22A | 0.514151151 | -0.499134185 | -0.623078937 |
| 5910463 | PKIA | 0.768647243 | -0.59790896 | -0.605146359 |
| 1190138 | TXK | 0.746973129 | -0.650777881 | -0.629179438 |
| 3390484 | SERINC2 | 0.776206414 | 0.64886317 | 0.808256073 |
| 2760148 | IL11RA | 0.8193691 | -0.78182714 | -0.660744815 |
| 5220070 | HLA-F | 0.863202757 | -0.663979001 | -0.751355428 |
| 3610288 | LAT | 0.615401662 | -0.725031406 | -0.478861361 |
| 460259 | LAT | 0.734031236 | -0.820491731 | -0.544466492 |
| 7050519 | CLEC4D | 0.628150633 | 0.690704651 | 0.432119284 |
| 7210253 | LOC730415 | 0.811244366 | -0.826115122 | -0.717588653 |
| 4480279 |  | 0.554543446 | -0.688747815 | -0.712991546 |
| 2340327 | CD320 | 0.846531861 | -0.598473495 | -0.617581186 |
| 2340703 | LOC100133372 | 0.164015207 | -0.565295594 | -0.411309398 |
| 5360364 | TAGAP | 0.58956957 | -0.685202199 | -0.600253759 |
| 1820379 | PTGDS | 0.889350449 | -0.627296796 | -0.610023464 |
| 1570348 | CCND2 | 0.798090303 | -0.616297962 | -0.490720522 |
| 4290209 | TMC6 | 0.566248993 | -0.654142814 | -0.453952661 |
| 1030743 | LTA | 0.873901419 | -0.709613588 | -0.727185783 |
| 160736 | MRI1 | 0.704225067 | -0.582456497 | -0.441935737 |
| 2750575 |  | 0.885490774 | -0.708863465 | -0.686181846 |
| 1300500 | ZNF827 | 0.698240391 | -0.667931559 | -0.610988267 |
| 4590477 | MAP4K1 | 0.850299223 | -0.849146586 | -0.668886571 |
| 1470184 | CLSTN1 | 0.843699616 | -0.763718522 | -0.62157607 |
| 5050347 | CD5 | 0.86308763 | -0.718274297 | -0.660264373 |
| 6590131 | HP | 0.85734137 | 0.721404359 | 0.550109133 |
| 160019 | SORT1 | 0.668300292 | 0.669187237 | 0.583008602 |
| 3440754 | IL32 | 0.898456895 | -0.764711879 | -0.574403655 |
| 6180176 | AES | 0.517313868 | -0.793808126 | -0.678892891 |
| 2190019 | CD160 | 0.788591665 | -0.665468161 | -0.742076513 |
| 5390730 | PIK3IP1 | 0.90048896 | -0.674307492 | -0.625886658 |
| 3360615 | FCER1A | 0.904418413 | -0.657086399 | -0.712440721 |
| 7330523 | C5orf39 | 0.874637394 | -0.66858555 | -0.694492175 |
| 2060482 | KIAA0907 | 0.731604369 | -0.574843356 | -0.548382916 |
| 5270753 | ARG1 | 0.739246382 | 0.827970356 | 0.593041169 |
| 3290731 | PRKCH | 0.87603161 | -0.675095396 | -0.750717109 |
| 1660681 | CCDC102A | 0.799537616 | -0.559213521 | -0.614148807 |
| 4250630 | KLRF1 | 0.827587544 | -0.628004298 | -0.679373831 |
| 3460520 | CCND2 | 0.809221251 | -0.564564838 | -0.534619992 |
| 5720373 | ANO9 | 0.742981512 | -0.647292393 | -0.629352183 |
| 4850538 | PAOX | 0.746275468 | -0.640900458 | -0.577726723 |
| 3450154 | TRAF3IP3 | 0.809483763 | -0.68726322 | -0.81086584 |
| 160070 | C16orf30 | 0.82027231 | -0.732292788 | -0.763547854 |
| 2650564 | RARRES3 | 0.933356312 | -0.754823077 | -0.781216256 |
| 7380689 | RPL22 | 0.776453391 | -0.719008359 | -0.732099968 |
| 3370242 | DKFZp761P0423 | 0.923923007 | -0.649403118 | -0.733353253 |
| 6180408 | NOSIP | 0.718471121 | -0.672325053 | -0.614024789 |
| 5310224 | LOC100133678 | 0.949681481 | -0.772995368 | -0.764527156 |
| 2360719 | IRAK3 | 0.779227559 | 0.808123184 | 0.680371841 |
| 5870192 | SKAP1 | 0.868751198 | -0.738530624 | -0.735219196 |
| 1770598 | KLRD1 | 0.839299763 | -0.589212718 | -0.735229171 |
| 6370435 | ETS1 | 0.871370838 | -0.554040637 | -0.626801289 |
| 240333 | ETS1 | 0.862250363 | -0.625711564 | -0.713668504 |
| 5050193 | RLTPR | 0.748311325 | -0.599854825 | -0.70687617 |
| 2710754 | CD96 | 0.908508474 | -0.699817412 | -0.680248093 |
| 4210619 | CD2 | 0.92269495 | -0.761709179 | -0.700066113 |
| 3400538 | IGFBP7 | 0.717132554 | 0.624108613 | 0.586289818 |
| 7160612 | USP36 | 0.719411095 | -0.630684446 | -0.632033319 |
| 2480128 | FBXO21 | 0.646240092 | -0.671906566 | -0.667656977 |
| 1980184 | P2RY10 | 0.841482465 | -0.666133323 | -0.738183919 |
| 3830471 | KIAA0748 | 0.916957702 | -0.661217153 | -0.591202272 |
| 7550358 | NELL2 | 0.912063784 | -0.694468391 | -0.739270021 |
| 4900086 | HAPLN3 | 0.801795991 | -0.664318941 | -0.546087093 |
| 2850021 | QSOX1 | 0.872527625 | 0.681806896 | 0.7617337 |
| 5360079 | GIMAP5 | 0.866609735 | -0.763418306 | -0.649131039 |
| 2260241 | LPAR5 | 0.641235281 | -0.746922652 | -0.73155535 |
| 2810064 | BTLA | 0.635996259 | -0.522987099 | -0.647432455 |
| 3170128 | CD8A | 0.918020279 | -0.701571125 | -0.660606137 |
| 670369 | CD8A | 0.940531872 | -0.715890308 | -0.674702015 |
| 6110736 | IRS2 | 0.26212804 | 0.553825446 | 0.440024746 |
| 4490612 | CYB561D1 | 0.560583424 | -0.623682302 | -0.479862294 |
| 770538 | LYSMD2 | 0.70598411 | -0.620015663 | -0.616663204 |
| 270338 | TRAT1 | 0.756388909 | -0.659251846 | -0.772247541 |
| 3890400 | CXCR5 | 0.806928721 | -0.654715158 | -0.582423155 |
| 1050082 | KIAA1147 | 0.930784818 | -0.690849036 | -0.711287204 |
| 4490520 | EBI2 | 0.784941549 | -0.594464782 | -0.527830838 |
| 2640341 | FKBP5 | 0.924232393 | 0.745569362 | 0.733819239 |
| 360402 | AMPH | 0.897643118 | 0.623585563 | 0.663713806 |
| 5860050 | LOC388564 | 0.593139582 | -0.699888113 | -0.633738226 |
| 6900458 | LOC728481 | 0.785485938 | -0.608110321 | -0.467349922 |
| 1710736 | DOCK10 | 0.742817085 | -0.649458825 | -0.742536014 |
| 830440 | TLR5 | 0.406767683 | 0.617003688 | -0.010513991 |
| 3840554 | SPOCK2 | 0.934642327 | -0.74596508 | -0.631894096 |
| 1260482 | GZMK | 0.898684688 | -0.684881951 | -0.694587808 |
| 2970408 | CD8A | 0.849700561 | -0.646141481 | -0.597214543 |
| 3840215 | FAM102A | 0.887159743 | -0.755085461 | -0.714833056 |
| 1850546 | FAM102A | 0.867139317 | -0.692399786 | -0.705125325 |
| 4200541 | FAM113B | 0.928815089 | -0.796371707 | -0.734708207 |
| 3930541 | PLEKHA1 | 0.790553025 | -0.684880522 | -0.681934173 |
| 3170091 | GIMAP7 | 0.582419693 | -0.756581703 | -0.618943759 |
| 5130440 | BIN1 | 0.865681538 | -0.78738933 | -0.61394352 |
| 7160474 | HLA-DQB1 | 0.942421802 | -0.715653834 | -0.665332265 |
| 1300192 | ARL4C | 0.708469309 | -0.624141167 | -0.659623174 |
| 5270544 | GRAP | 0.899887799 | -0.731254277 | -0.733773748 |
| 6290400 | CD247 | 0.94569796 | -0.733267211 | -0.692016996 |
| 1070367 | C19orf59 | 0.846856939 | 0.788668001 | 0.671544886 |
| 6020523 | PPP2R2B | 0.875434867 | -0.613501528 | -0.611116808 |
| 7150689 | LOC644907 | 0.496714899 | -0.475641139 | -0.423975793 |
| 5220612 | ECHDC3 | 0.709361863 | 0.688595578 | 0.499596462 |
| 6550754 | EVL | 0.905540584 | -0.736561211 | -0.671982813 |
| 4250008 | ATM | 0.635949452 | -0.589579319 | -0.803993631 |
| 3800168 | SLC2A3 | 0.49492992 | 0.720967673 | 0.537737441 |
| 1940373 | ZNF831 | 0.678501082 | -0.638249898 | -0.590298179 |
| 6270040 | ZNF831 | 0.658285539 | -0.704332913 | -0.673677392 |
| 1580576 | ITM2A | 0.837597737 | -0.786627587 | -0.777799379 |
| 6200019 | KLRB1 | 0.912085258 | -0.678123921 | -0.715505458 |
| 670255 | GADD45A | 0.763779437 | 0.764737891 | 0.550353806 |
| 4880673 | GADD45A | 0.847680387 | 0.775323865 | 0.586945566 |
| 290022 |  | 0.810324455 | -0.665103656 | -0.633633794 |
| 4860634 | CCM2 | 0.581868994 | -0.649893773 | -0.74577085 |
| 4260725 | CD79B | 0.77560374 | -0.676365135 | -0.665428739 |
| 1410730 | VSIG4 | 0.932986557 | 0.76851099 | 0.815406617 |
| 6420392 | OCIAD2 | 0.849223993 | -0.701636291 | -0.710305625 |
| 2140382 | PASK | 0.827130867 | -0.72547224 | -0.585236179 |
| 3800019 | NCR3 | 0.780601753 | -0.656340498 | -0.651130062 |
| 70386 | CD8B | 0.823777569 | -0.650533031 | -0.564403563 |
| 6650324 | PLEKHA1 | 0.795681359 | -0.621378142 | -0.725002574 |
| 3520603 | LOC440311 | 0.599089218 | -0.697651537 | -0.545509861 |
| 3140041 | SP140 | 0.784735753 | -0.684576749 | -0.6543381 |
| 5310053 | LTB | 0.792493032 | -0.683991778 | -0.659009456 |
| 5420091 | LTB | 0.70938872 | -0.811281674 | -0.728392052 |
| 1710630 | ST6GAL1 | 0.776480002 | -0.71571168 | -0.796777716 |
| 5570180 | LCK | 0.823514254 | -0.652478661 | -0.634044522 |
| 6860240 |  | 0.918567477 | -0.781796556 | -0.717301995 |
| 3610309 | LOC653881 | 0.720998295 | -0.613336359 | -0.55462627 |
| 4610674 | BTN3A2 | 0.939387082 | -0.71695198 | -0.778168773 |
| 3930326 | LOC728014 | 0.759799933 | -0.643708928 | -0.756632332 |
| 3940041 | SIRPG | 0.766822027 | -0.752383272 | -0.682148067 |
| 2480068 | CD63 | 0.868448058 | 0.770055013 | 0.740039407 |
| 4780224 | STMN3 | 0.941222261 | -0.736109607 | -0.74823803 |
| 3170703 | LY9 | 0.88456784 | -0.745642179 | -0.747401434 |
| 6550243 | ESYT1 | 0.802925257 | -0.723155129 | -0.664022808 |
| 2470161 | PLEKHF1 | 0.780212064 | -0.670624982 | -0.619631224 |
| 6180133 | ITPR3 | 0.864867512 | -0.711828005 | -0.668655135 |
| 3890689 | CD247 | 0.946165743 | -0.751790432 | -0.682490078 |
| 6770309 | MYL6 | 0.83991354 | 0.6329994 | 0.587127328 |
| 1410603 | RAB3IP | 0.614694435 | -0.565604971 | -0.347086325 |
| 7510482 | RPS4X | 0.625055504 | -0.594135109 | -0.580368325 |
| 6040563 | RPS4X | 0.831615913 | -0.579907838 | -0.457136204 |
| 3840471 | LOC728782 | -0.125076847 | -0.09104139 | -0.68023644 |
| 1780440 | CD79A | 0.639459867 | -0.563474722 | -0.607233571 |
| 4120224 | GPR18 | 0.573569198 | -0.682845043 | -0.698114788 |
| 6330091 | CD7 | 0.893783197 | -0.756667874 | -0.696115409 |
| 1260156 | LOC654194 | 0.048183034 | -0.114832778 | -0.616875023 |
| 1990079 | FBXO32 | 0.825831948 | -0.606660555 | -0.618076016 |
| 5490546 | SLC30A1 | 0.358888814 | 0.785540598 | 0.566052565 |
| 1400689 | JAM3 | 0.698231322 | -0.539929761 | -0.570720761 |
| 6860753 | TSPO | 0.755547721 | 0.712830223 | 0.577730121 |
| 3890523 | IL7R | 0.915407332 | -0.705595808 | -0.636430633 |
| 1170671 | CD3D | 0.900297758 | -0.655482069 | -0.651385422 |
| 1450471 | LOC202134 | 0.875161736 | -0.601099856 | -0.635666274 |
| 150400 | LBA1 | 0.691235189 | -0.696798598 | -0.664876085 |
| 50706 | CD40LG | 0.768152687 | -0.661443872 | -0.687333965 |
| 1190626 | ZMYND11 | 0.758174104 | -0.556361645 | -0.617157408 |
| 290750 | SAMD3 | 0.868184828 | -0.64206352 | -0.695715744 |
| 1090474 | CD3G | 0.727175079 | -0.566946499 | -0.708144854 |
| 6960397 | EPHX2 | 0.85612643 | -0.700090689 | -0.689258625 |
| 1230022 | LOC642817 | 0.270759064 | -0.521226102 | -0.391753518 |
| 4760338 | CDC25B | 0.821597837 | -0.665701068 | -0.553272556 |
| 540747 |  | 0.806889741 | -0.633202296 | -0.737672486 |
| 6200670 | LPIN1 | 0.790828318 | -0.61633553 | -0.785294049 |
| 4920070 | RPL21 | 0.132903067 | -0.033485226 | -0.645720068 |
| 7000270 | PYHIN1 | 0.856633137 | -0.645552624 | -0.678810713 |
| 580411 | LAX1 | 0.863946011 | -0.72760438 | -0.642332529 |
| 4200725 | HLA-DRB6 | 0.901730416 | -0.787665341 | -0.777415771 |
| 1850523 | GZMB | 0.938782663 | -0.644522272 | -0.665799966 |
| 3180468 | LOC286208 | 0.669960866 | -0.631911335 | -0.586974033 |
| 6380672 | CA4 | 0.389864135 | 0.644039533 | 0.351676999 |
| 6270020 |  | 0.668786668 | -0.810533882 | -0.570364388 |
| 4150189 | CTSL1 | 0.866056728 | 0.737092277 | 0.744945823 |
| 3850767 | SBK1 | 0.91007882 | -0.74649751 | -0.708283059 |
| 2650706 | TRADD | 0.840659285 | -0.699217031 | -0.720213783 |
| 5390246 | CCR7 | 0.918032965 | -0.634736364 | -0.624633694 |
| 160672 | TCF7 | 0.829417583 | -0.618280534 | -0.650650667 |
| 1440398 | LOC644511 | 0.75098009 | -0.592364109 | -0.683891362 |
| 3850411 | LOC653316 | 0.834087315 | -0.728232991 | -0.61998427 |
| 5720768 |  | 0.57874863 | 0.72988629 | 0.488892798 |
| 1240450 | CD27 | 0.923030548 | -0.778596992 | -0.725407822 |
| 6280750 |  | 0.916888723 | -0.727450997 | -0.640834549 |
| 6380364 | SLC7A6 | 0.666548229 | -0.635869329 | -0.602587586 |
| 7200743 | SLAMF6 | 0.898670844 | -0.738570743 | -0.667308608 |
| 5270520 | FAIM3 | 0.937354139 | -0.740611034 | -0.721838517 |
| 5050689 | PLD1 | 0.755102297 | 0.672222055 | 0.672581803 |
| 830324 | FLT3LG | 0.880828585 | -0.814392777 | -0.769672743 |
| 940519 | GPR44 | 0.656759217 | -0.527090131 | -0.41403626 |
| 3940438 | NCF1 | 0.232968486 | -0.626607863 | -0.252648584 |
| 3420612 | GZMA | 0.899215116 | -0.629119449 | -0.70185693 |
| 1190039 | HLA-DPA1 | 0.880380081 | -0.820589375 | -0.797939427 |
| 1440750 | S1PR5 | 0.909702159 | -0.637828488 | -0.676307846 |
| 1500703 | DENND2D | 0.79673072 | -0.790841743 | -0.735931116 |
| 3990224 | TNFRSF25 | 0.859583608 | -0.730567598 | -0.718186714 |
| 2650274 | C19orf60 | 0.497287036 | -0.737154359 | -0.579373562 |
| 130161 | LCK | 0.803686422 | -0.747499861 | -0.669943116 |
| 6960168 | ATP8B2 | 0.875864447 | -0.753009955 | -0.742415028 |
| 4560743 | CD96 | 0.792420464 | -0.657082136 | -0.633157934 |
| 2070451 | PAQR8 | 0.834118308 | -0.547733963 | -0.498193447 |
| 1400274 | IL32 | 0.90108819 | -0.689891384 | -0.62556365 |
| 4390315 | LOC645436 | 0.346298676 | -0.618476165 | -0.399298777 |
| 4780678 | ZAP70 | 0.858468107 | -0.779046999 | -0.648295499 |
| 7330538 | PYHIN1 | 0.925776824 | -0.665800803 | -0.67348126 |
| 2640441 | PRAGMIN | 0.889548921 | -0.728119501 | -0.72362783 |
| 4890241 | GPR56 | 0.913592679 | -0.663296066 | -0.651049668 |
| 460113 | MAGED1 | 0.822627179 | -0.685458631 | -0.518218334 |
| 3840288 | LRFN3 | 0.76997394 | -0.669294965 | -0.693586267 |
| 2370041 | LRRN3 | 0.912094809 | -0.620713883 | -0.573690244 |
| 5490068 | MCOLN2 | 0.811444225 | -0.554694546 | -0.666316472 |
| 1430717 | BTN3A3 | 0.816556509 | -0.748657522 | -0.766123866 |
| 1980524 | GBP4 | 0.93329757 | -0.567031132 | -0.521336528 |
| 4260139 | AKR1B1 | 0.674659806 | -0.786959064 | -0.490886757 |
| 4760053 | C2orf89 | 0.89826036 | -0.695824713 | -0.729840187 |
| 4040088 | TCP11L2 | 0.835587916 | 0.624799611 | 0.441389561 |
| 4280044 | LOC100131609 | 0.15661074 | -0.558926921 | -0.393081766 |
| 7650440 | BACH2 | 0.775507155 | -0.608069611 | -0.592525399 |
| 1030376 | PLCG1 | 0.794589762 | -0.547069141 | -0.513831927 |
| 2260131 | PLCG1 | 0.879265465 | -0.650769825 | -0.698268748 |
| 1690044 | CA5B | 0.690465587 | -0.343140449 | -0.441142705 |
| 6590228 | CD6 | 0.915354257 | -0.75982616 | -0.683241261 |
| 1780600 | CD3E | 0.785131005 | -0.641610972 | -0.707083334 |
| 1450671 | CARD11 | 0.819963356 | -0.765199313 | -0.721589471 |
| 6180088 | ATP6V0E2 | 0.835492605 | -0.746117616 | -0.651506526 |
| 3390594 | KLRD1 | 0.888577702 | -0.668745866 | -0.682623422 |
| 2260196 | LOC392437 | 0.783014086 | 0.613951139 | 0.67862796 |
| 60093 | NCALD | 0.82786299 | -0.645454779 | -0.673878911 |
| 4200452 | HLA-DOA | 0.805068063 | -0.683060005 | -0.666637996 |
| 6860678 | HNRPDL | 0.701572053 | -0.603224816 | -0.446670266 |
| 3930537 |  | 0.806621249 | -0.6167741 | -0.705786348 |
| 7570408 | CCL5 | 0.910580021 | -0.805085674 | -0.747301037 |
| 620717 | CCL5 | 0.910941122 | -0.854084579 | -0.817044541 |
| 6180148 | MAFG | 0.60324416 | 0.756468052 | 0.691620719 |
| 1580411 | CD3D | 0.897552335 | -0.625685834 | -0.667986788 |
| 730156 | ICAM2 | 0.778700828 | -0.700959828 | -0.482870601 |
| 2350091 | LPXN | 0.63931275 | -0.699002549 | -0.60509313 |
| 1410221 | S100A12 | 0.770851119 | 0.680960586 | 0.42860956 |
| 2850600 | TMEM204 | 0.844072299 | -0.802770244 | -0.782233158 |
| 4860242 | TAGAP | 0.529940434 | -0.614980839 | -0.588315253 |
| 4040187 | C6orf190 | 0.744002286 | -0.608034203 | -0.683780658 |
| 2810615 | GRB10 | 0.84900094 | 0.79983679 | 0.796429717 |
| 6180228 | FCRL3 | 0.897086896 | -0.608346099 | -0.645913803 |
| 6130725 | ARHGEF18 | 0.691336123 | -0.687079054 | -0.568113842 |
| 6450594 | CD79B | 0.801298141 | -0.695174433 | -0.707378745 |
| 7050619 | LRRFIP2 | 0.74766318 | 0.706923994 | 0.621451422 |
| 3930382 | NMT2 | 0.878645346 | -0.655242545 | -0.750701903 |
| 510079 | HLA-DRB4 | 0.786896097 | -0.647256012 | -0.476489649 |
| 780403 | HLA-DQA1 | 0.952408559 | -0.722527652 | -0.733863489 |
| 4920328 | SH3KBP1 | 0.763035092 | -0.657854677 | -0.593099818 |
| 240494 | TCF7 | 0.821080725 | -0.688199434 | -0.623790266 |
| 5810743 | LOC387882 | 0.764851255 | -0.655464577 | -0.791643324 |
| 2810400 | KLHL3 | 0.798490129 | -0.679661046 | -0.693710803 |
| 840025 | FYN | 0.855927853 | -0.724096955 | -0.646739555 |
| 6400603 | PVRIG | 0.885611979 | -0.684578148 | -0.765516371 |
| 5490333 | PPP1R16B | 0.766275643 | -0.681731394 | -0.765375626 |
| 4540241 | C5orf32 | 0.796869621 | 0.696595003 | 0.727828157 |
| 4880193 | KLRG1 | 0.902950288 | -0.608480462 | -0.653940144 |
| 6550600 | MYC | 0.742328745 | -0.532474938 | -0.587249205 |
| 3780181 | FLOT1 | 0.31594482 | 0.584482754 | 0.63731004 |
| 5690671 | GOLGA8B | 0.803742052 | -0.594237519 | -0.672871512 |
| 7320307 | PDCD7 | 0.562555702 | -0.607093524 | -0.538027104 |
| 5910465 | SH2D1A | 0.848923082 | -0.763976061 | -0.769646764 |
| 1510673 | LOC644132 | 0.816827775 | 0.772691164 | 0.688939372 |
| 5490768 | GPR56 | 0.909004298 | -0.645956012 | -0.665613309 |
| 6420450 | LIME1 | 0.860233704 | -0.811825729 | -0.718751879 |
