# Supplementary material for: Comparison of Whole Blood and Peripheral Blood Mononuclear Cell Gene Expression for Evaluation of the Perioperative Inflammatory Response in Patients with Advanced Heart Failure
Source: PLoS One. 2014 Dec 17;9(12):e115097. doi: 10.1371/journal.pone.0115097 (PMC4269402; doi:10.1371/journal.pone.0115097)
Supplement: S3 Table — GO categories overlapping between PBMC and WB after intervention. (DOCX) [file pone.0115097.s003.docx]

**Table S3.** GO categories overlapping between PBMC and WB after intervention.

| **GO accession** | **GO Term** | **FDR** | **N**  **selection** | **% selection** | **N**  **Total** | **% Total** |
| --- | --- | --- | --- | --- | --- | --- |
| GO:0001775 | cell activation | 0.053 | 55 | 2.0841227 | 208 | 1.244 |
| GO:0001817 | regulation of cytokine production | 0.061 | 37 | 1.4020462 | 125 | 0.747 |
| GO:0002376 | immune system process | 0.000 | 243 | 9.208034 | 823 | 4.921 |
| GO:0002520 | immune system development | 0.008 | 56 | 2.122016 | 196 | 1.172 |
| GO:0002682 | regulation of immune system process | 0.000 | 85 | 3.220917 | 257 | 1.537 |
| GO:0002684 | positive regulation of immune system process | 0.002 | 50 | 1.894657 | 160 | 0.957 |
| GO:0002694 | regulation of leukocyte activation | 0.010 | 36 | 1.3641531 | 109 | 0.652 |
| GO:0002696 | positive regulation of leukocyte activation | 0.035 | 26 | 0.9852217 | 74 | 0.443 |
| GO:0005515 | protein binding | 0.000 | 1229 | 46.57067 | 6973 | 41.697 |
| GO:0005829 | Cytosol | 0.000 | 226 | 8.56385 | 879 | 5.256 |
| GO:0006915 | Apoptosis | 0.011 | 98 | 3.713528 | 425 | 2.541 |
| GO:0006917 | induction of apoptosis | 0.012 | 58 | 2.1978023 | 214 | 1.280 |
| GO:0006952 | defense response | 0.000 | 143 | 5.4187193 | 517 | 3.092 |
| GO:0006955 | immune response | 0.000 | 167 | 6.3281546 | 561 | 3.355 |
| GO:0008219 | cell death | 0.012 | 109 | 4.1303525 | 483 | 2.888 |
| GO:0010941 | regulation of cell death | 0.000 | 135 | 5.115574 | 545 | 3.259 |
| GO:0010942 | positive regulation of cell death | 0.002 | 72 | 2.7283063 | 270 | 1.615 |
| GO:0012501 | programmed cell death | 0.010 | 99 | 3.751421 | 429 | 2.565 |
| GO:0012502 | induction of programmed cell death | 0.013 | 58 | 2.1978023 | 215 | 1.286 |
| GO:0019955 | cytokine binding | 0.008 | 35 | 1.32626 | 103 | 0.616 |
| GO:0042101 | T cell receptor complex | 0.000 | 11 | 0.41682455 | 12 | 0.072 |
| GO:0042981 | regulation of apoptosis | 0.000 | 135 | 5.115574 | 537 | 3.211 |
| GO:0043065 | positive regulation of apoptosis | 0.002 | 71 | 2.690413 | 265 | 1.585 |
| GO:0043067 | regulation of programmed cell death | 0.000 | 135 | 5.115574 | 544 | 3.253 |
| GO:0043071 | positive regulation of programmed cell death | 0.003 | 71 | 2.690413 | 268 | 1.603 |
| GO:0043235 | receptor complex | 0.021 | 30 | 1.1367942 | 88 | 0.526 |
| GO:0045058 | T cell selection | 0.048 | 9 | 0.3410383 | 14 | 0.084 |
| GO:0045321 | leukocyte activation | 0.014 | 51 | 1.9325502 | 178 | 1.064 |
| GO:0046649 | lymphocyte activation | 0.006 | 46 | 1.7430845 | 149 | 0.891 |
| GO:0048518 | positive regulation of biological process | 0.027 | 296 | 11.21637 | 1521 | 9.095 |
| GO:0050863 | regulation of T cell activation | 0.050 | 28 | 1.061008 | 84 | 0.502 |
| GO:0050865 | regulation of cell activation | 0.010 | 37 | 1.4020462 | 113 | 0.676 |
| GO:0050867 | positive regulation of cell activation | 0.035 | 26 | 0.9852217 | 74 | 0.443 |
| GO:0051249 | regulation of lymphocyte activation | 0.040 | 32 | 1.2125806 | 100 | 0.598 |
| GO:0051251 | positive regulation of lymphocyte activation | 0.065 | 24 | 0.9094354 | 69 | 0.413 |
